# A window into eye movement dysfunction following mTBI: A scoping review of magnetic resonance imaging and eye tracking findings

**DOI:** 10.1002/brb3.2714

**Published:** 2022-07-21

**Authors:** Matthew A. McDonald, Maryam Tayebi, Joshua P. McGeown, Eryn E. Kwon, Samantha J Holdsworth, Helen V Danesh‐Meyer

**Affiliations:** ^1^ Department of Ophthalmology University of Auckland Auckland New Zealand; ^2^ Mātai Medical Research Institute Gisborne New Zealand; ^3^ Auckland Bioengineering Institute University of Auckland Auckland New Zealand; ^4^ Auckland University of Technology Traumatic Brain Injury Network Auckland New Zealand; ^5^ Department of Anatomy and Medical Imaging University of Auckland Auckland New Zealand; ^6^ Eye Institute Auckland New Zealand

**Keywords:** concussion, DTI, eye tracking, fMRI, MRI, mTBI, ocular motor, oculomotor, saccades, smooth pursuit, white matter tracts

## Abstract

Mild traumatic brain injury (mTBI), commonly known as concussion, is a complex neurobehavioral phenomenon affecting six in 1000 people globally each year. Symptoms last between days and years as microstructural damage to axons and neurometabolic changes result in brain network disruption. There is no clinically available objective biomarker to diagnose the severity of injury or monitor recovery. However, emerging evidence suggests eye movement dysfunction (e.g., saccades and smooth pursuits) in patients with mTBI. Patients with a higher symptom burden and prolonged recovery time following injury may show higher degrees of eye movement dysfunction. Likewise, recent advances in magnetic resonance imaging (MRI) have revealed both white matter tract damage and functional network alterations in mTBI patients, which involve areas responsible for the ocular motor control. This scoping review is presented in three sections: Section 1 explores the anatomical control of eye movements to aid the reader with interpreting the discussion in subsequent sections. Section 2 examines the relationship between abnormal MRI findings and eye tracking after mTBI based on the available evidence. Finally, Section 3 communicates gaps in our knowledge about MRI and eye tracking, which should be addressed in order to substantiate this emerging field.

## Graphical Abstract

This review aims to scientifically resolve the correlation between eye movement dysfunction in mTBI with advanced neuroimaging. Included are the neuroanatomical basis of eye movement control, novel MRI correlations to eye tracking measures in mTBI patients, and areas requiring future research.

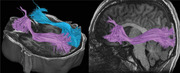

## 1 INTRODUCTION

Traumatic brain injury is common, affecting over 50 million people per year worldwide (James et al., 2019; Rusnak, 2013). Mild traumatic brain injury (mTBI or concussion) consists of up to 95% of these injuries and is regarded as a silent epidemic (Rusnak, 2013). This condition is non‐life threatening but can significantly impact quality of life. Prolonged symptom burden has been cited from 30% to 55% in patients after 2 weeks (Barker‐Collo et al., 2016; Kara et al., 2020) and 48% (Theadom et al., 2016) at 1‐year follow‐up. The elderly are particularly at high risk with worse prognosis and early cognitive decline (Goldstein et al., 1999; Rapoport & Feinstein, 2000).

This injury is considered to have two phases. During the primary phase, the brain undergoes discrete deformation due to an external biomechanical load (acceleration–deceleration)—both from direct or indirect impact (e.g., falls, blast exposure, motor vehicle collision, interpersonal violence, or sport‐related collision). Consequently, neuronal and vascular tissues are exposed to abrupt stretching and shearing forces, triggering the secondary phase of injury which is characterized by a complex neurophysiological cascade that evolves in the minutes, hours, and days post‐mTBI, as extrapolated from predominantly animal studies (Banks & Dominguez, 2019; Bigler & Maxwell, 2012; V. E. Johnson et al., 2013; Romeu‐Mejia et al., 2019; Toledo et al., 2012). Patients with mTBI experience functional disturbances in the form of clinical signs/symptoms and reduced quality of life as a result of these neurophysiological disruptions.

Despite an understanding of the pathophysiology of mTBI, an objective, reliable, and valid biomarker to guide clinical decision making has yet to be identified. This means clinicians are faced with the difficult task of diagnosing this injury, predicting which patients are at the highest risk of prolonged recovery and determining recovery based on subjective symptom reports and clinical examination. The occurrence of mTBI resulting from sport or physical activity (∼20% of mTBIs; Theadom et al., 2020) has garnered significant attention within the literature due to high participation rates in youth contact sports, propensity of athletes to under‐report their injuries, general desire of injured athletes to rapidly return to the activity that caused mTBI, repeated exposure to head impacts, and the proposed link between repeated head impacts and negative long‐term neurological outcomes. Cerebral vulnerability increases if repeated mTBI occurs prior to full recovery from the initial mTBI, which is particularly relevant to athletes playing collision sports (Prins et al., 2013). Accordingly, there is great need to identify an objective clinical biomarker to definitively diagnose mTBI, measure the effects of rehabilitation programs, and to determine sufficient neurophysiological recovery to safely return to education/work and sport.

**FIGURE 1:**
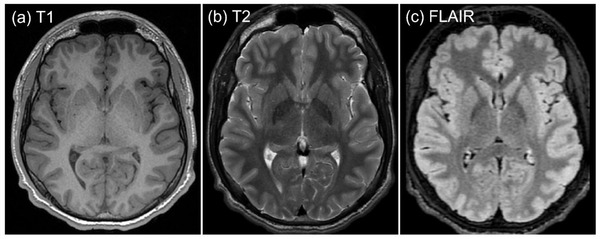
(a) T1‐weighted, (b) T2‐weighted, and (c) T2 FLAIR MRI sequences acquired on a healthy 17‐year‐old male, representing key sequences of a clinical MRI protocol

Utilization of medical imaging has become standard practice when assessing moderate and severe forms of traumatic brain injury. Computed tomography (CT) has become a vital tool in urgent care settings to exclude significant bleeds and pathology following moderate and severe traumatic brain injury requiring immediate neurosurgical intervention (e.g., skull fractures, clinically significant intracranial hemorrhages) (Toledo et al., 2012). Yet, CT offers minimal sensitivity (4–10%) to detect abnormalities useful in mTBI diagnosis, especially subtle microstructural and functional deficits (Borg et al., 2004; Culotta et al., 1996; Glass et al., 2015). Similarly, standard clinical brain magnetic resonance imaging (MRI) (e.g., T1, T2, and FLAIR sequence; Figure 1) demonstrates poor utility (0.7–30%) to detect mTBI (Klein et al., 2019; Mittl et al., 1994).

Despite the shortcomings of these widely used imaging techniques, more advanced MRI imaging techniques are being widely investigated as potential objective assessment methods of mTBI. Diffusion MRI (dMRI) and functional MRI (fMRI) are two imaging modalities receiving substantial attention in mTBI research initiatives due to improvements in both image acquisition and postprocessing methods.

**FIGURE 2:**
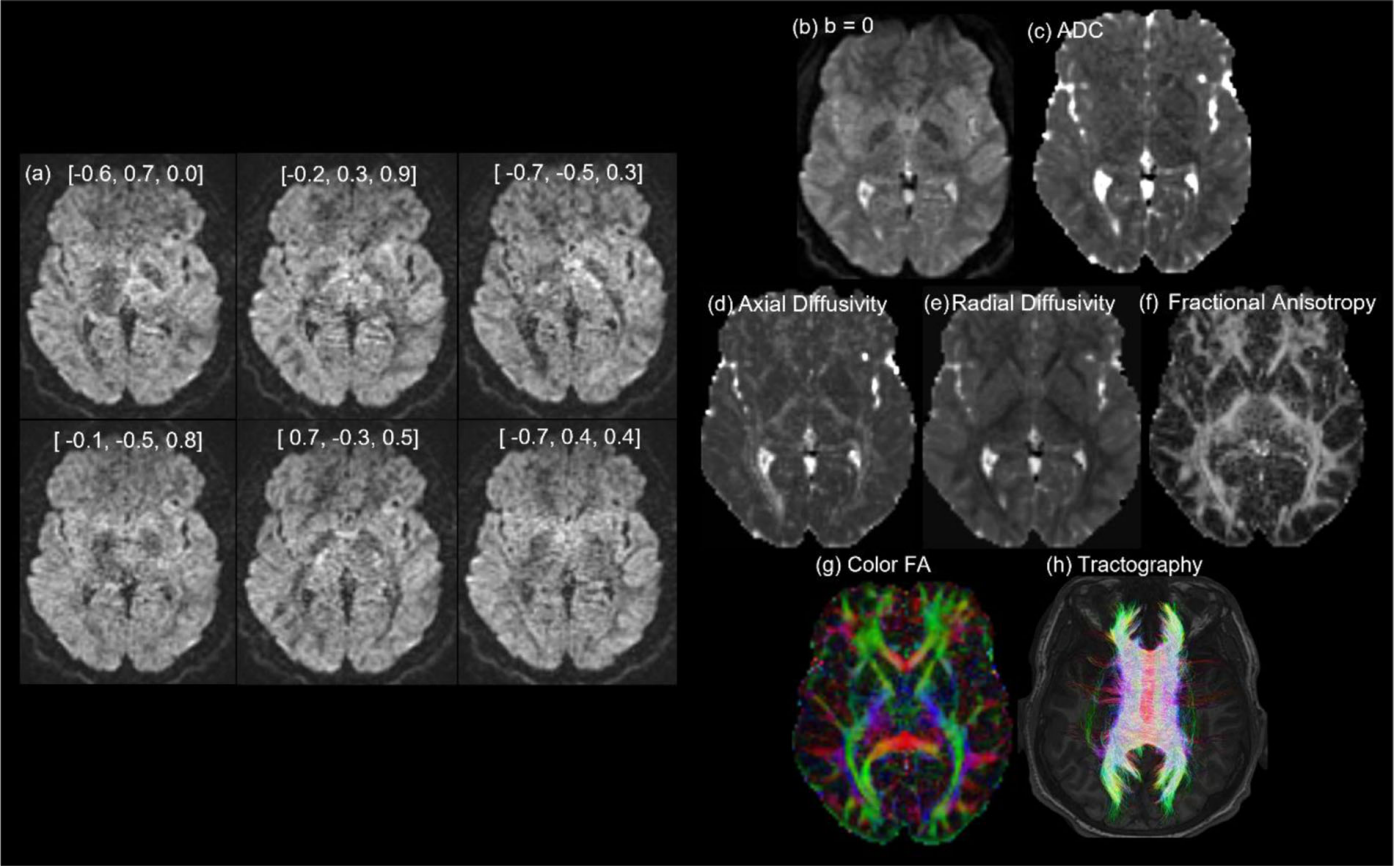
Diffusion MRI in a healthy 17‐year‐old male. To derive neural tract direction, DTI scans use six or more gradient directions, sufficient to compute the diffusion tensor. The level of diffusion weighting is indicated by the b‐value, a parameter that reflects the length and strength of the magnetic field gradients; slow moving water molecules across shorter diffusion distances require a higher b‐value (Stejskal & Tanner, 1965). (a) Six direction gradients used as input (directions in square brackets) which is combined with (b) to calculate diffusion parameters. (c) Apparent diffusion coefficient (ADC) map which requires a minimum of three directions. By collecting images with at least two different *b *values, a pure parametric image of the ADC can be calculated (Le Bihan, 2013), where the ADC represents the magnitude of diffusion of water molecules within the tissue as the sole source of contrast. (d) Axial diffusivity (AD). (e) Radial diffusivity (RD) map as a quantitative measurement of diffusion, removing T2 effects. (f) Fractional anisotropy (FA) map. (g) Color FA map. (h) Diffusion tractography map of corpus callosum with 54 diffusion gradients. *Note*: (d)–(g) require six or more directional gradients

**FIGURE 3:**
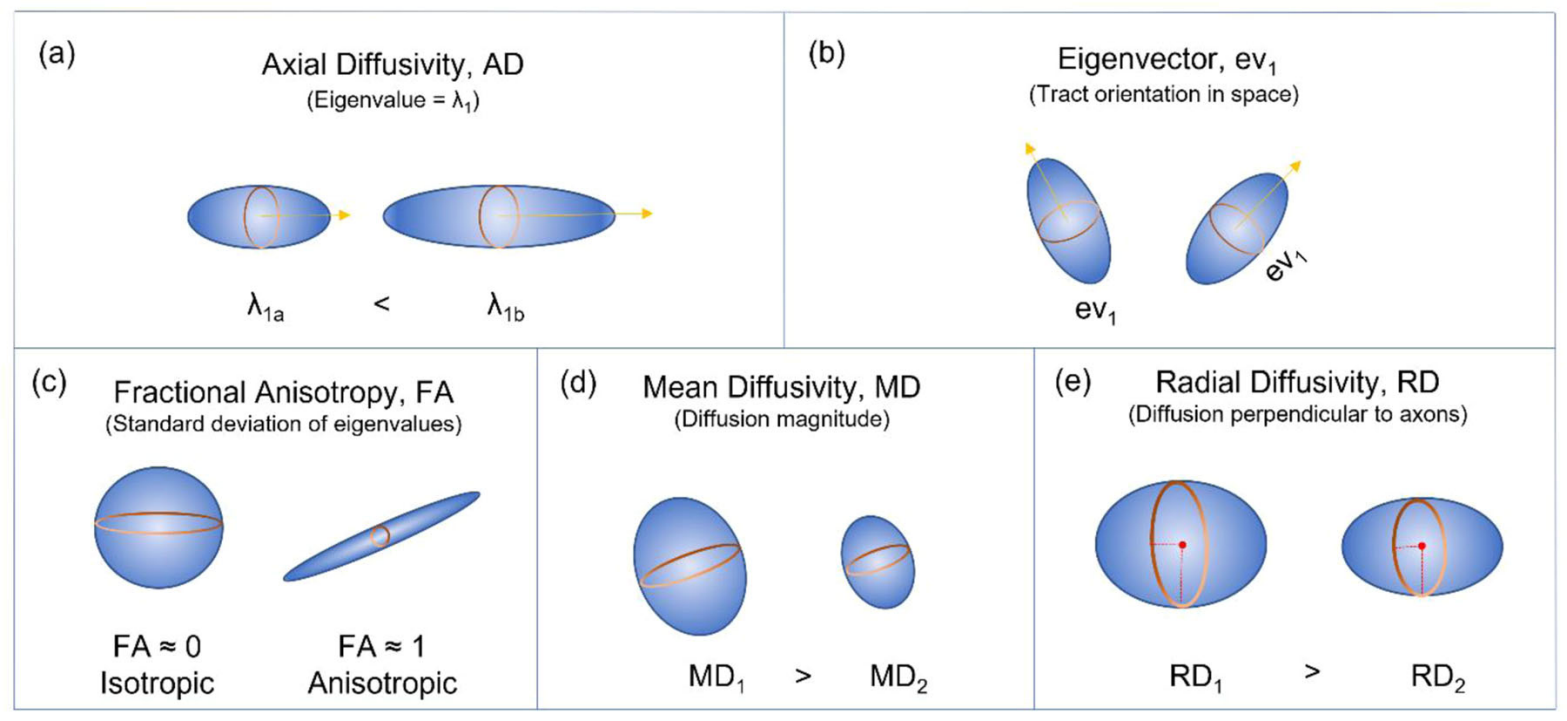
Values, which are eigenvalues (“pointiness” and “size” of diffusion) and eigenvectors (describing orientation in space), of the diffusion tensor are used to give directional information of water diffusion. (a)–(e) The five main diffusion MRI ellipsoid parameters to quantify water diffusion along a trajectory

Diffusion tensor imaging (DTI) is a form of dMRI scan, which provides contrast based on differences in the magnitude of diffusion of water molecules within the brain (Basser et al., 1994; Le Bihan, 2014; Le Bihan et al., 1986; Stejskal & Tanner, 1965). This reveals unique information of white matter microstructures to assist in the study of both axonal organization and disruption in the brain. Diffusion tractography is a 3D modeling technique used to reveal the white matter fiber trajectories using data collected by dMRI. Tractography visually represents the underlying structural connectivity of the brain and can be used inform the calculation of dMRI measurements used to examine mTBI pathophysiology. However, dMRI measures derived from tractography (Figures 2 and 3) rely on high‐quality images from the MRI, correct calculation of fiber direction per voxel, and application of the best‐suited tracking algorithm (Shawna et al., 2013). Shortcomings of tractography have been improved in recent years through the ability to resolve crossing white matter fibers through methods such as multitensor fitting (Tournier et al., 2004), Q‐ball imaging (Tuch et al., 2003), persistent angular structure MRI (Dell'Acqua & Tournier, 2019; Jansons & Alexander, 2003) and high angular resolution diffusion‐weighted imaging (HARDI) (Tuch, 1999). Delouche et al. (2016) provide a comprehensive summary of dMRI's role in mTBI.

**FIGURE 4:**
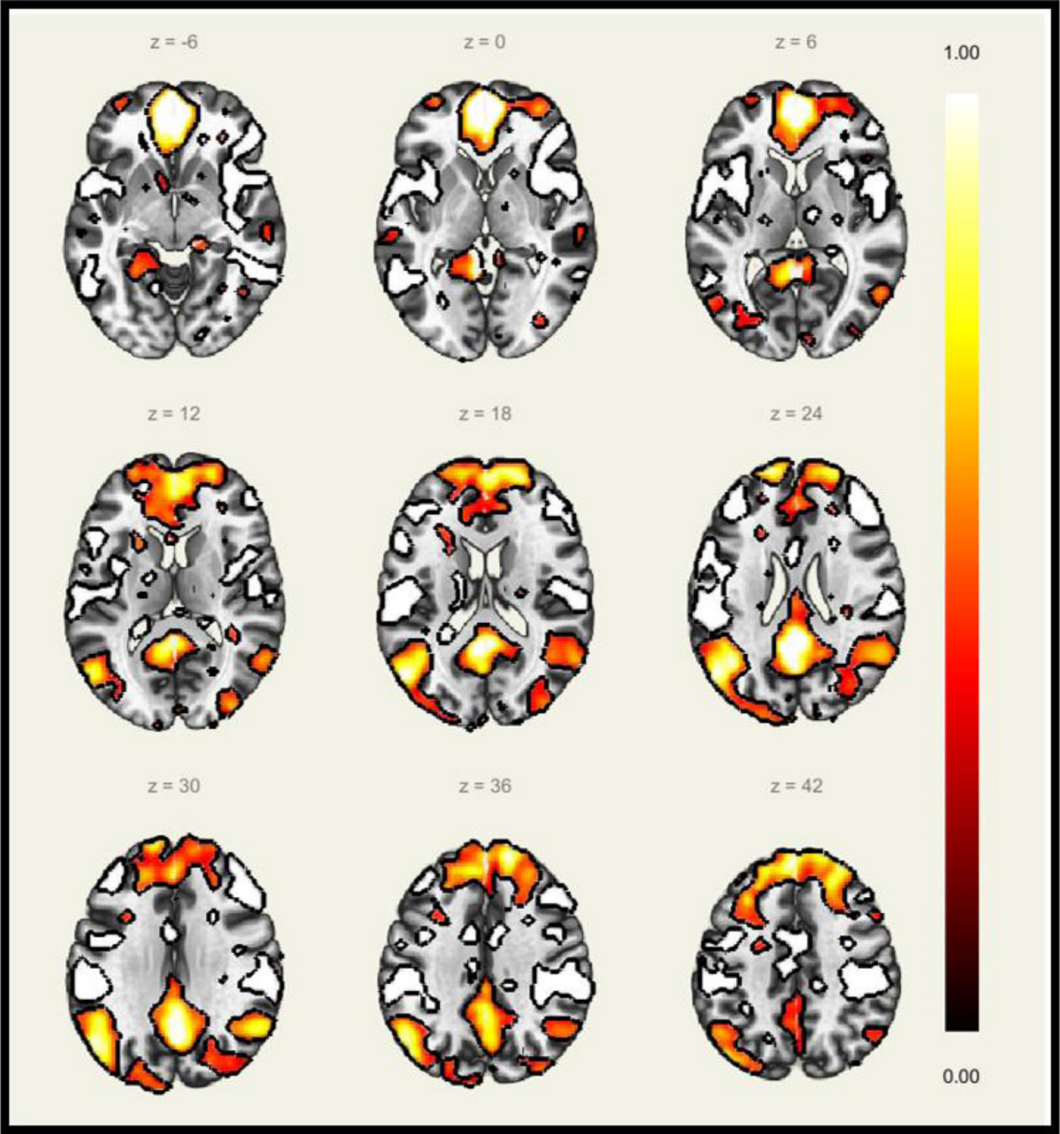
Default mode network BOLD signal activation in a 17‐year‐old male acquired on a resting state fMRI sequence while lying quietly awake with eyes closed. Resolution: 1.5 mm × 1.5 mm × 3 mm, acquired over 5 min. Ninety slices per location were recorded in a multiband sequence

fMRI indirectly measures brain activity by detecting changes associated with regional cerebral blood flow (Belliveau et al., 1991; Ogawa et al., 1990). This technique relies on the fact that cerebral blood flow and neuronal activation are coupled. When an area of the brain is in use, blood flow (i.e., the hemodynamic response) to that region increases in response to the increase in energy use by brain cells (Logothetis et al., 2001). This is measured via blood‐oxygen‐level dependent (BOLD) contrast, exploiting the variation in oxyhemoglobin to deoxyhemoglobin ratio at the site of neuronal activity in the brain (Ogawa et al., 1992) (Figure 4). Resting state fMRI (rs‐fMRI), or “taskless” fMRI, reveals a subject's baseline BOLD variance without stimuli (Biswal et al., 1995), while task‐based fMRI (requiring a task or stimuli presentation in the scanner) measures altered BOLD responses in different brain regions (Bandettini et al., 1992). Functional connectivity (FC), derived from BOLD signals, describes the temporal dependency of neuronal activation patterns of anatomically separated brain regions. For a comprehensive review of this area, we refer to Mayer et al. (2015). Altogether, the combination of dMRI and fMRI provides both structural (white matter) and functional (gray matter) information, facilitating our understanding of neuronal organization and subsequent dysfunction in mTBI.

Another promising method of objectively assessing mTBI is the evaluation of eye movements. Approximately 34% of mTBI patients present with symptoms of ocular motor dysfunction (Lumba‐Brown et al., 2019). However, subtle ocular motor abnormalities are hypothesized to be ubiquitous across clinical subtypes, which are typically classified via symptom clusters (Langdon et al., 2020; Lumba‐Brown et al., 2019). The Vestibular/Ocular Motor Screening Tool (VOMS) is used as a clinical assessment of gross oculomotor dysfunction post‐mTBI by assessing symptom provocation during smooth pursuits, saccades, convergence, vestibulo‐ocular reflex, and visual motion sensitivity (Mucha et al., 2014). While the VOMS is easy to implement clinically, it is scored subjectively and lacks the precision to detect more subtle alterations in ocular motor function such as velocities, latencies, accuracies (e.g., saccadic gain), or pupil parameters (e.g., constriction velocity and resting tone). In addition, symptom provocation yields high false positives (Knell et al., 2021) and does not provide objective evidence of dysfunction. Eye tracking technology offers precise measurement of these variables during the same functional movements evaluated during VOMS.

The control of eye movements involves widely distributed networks of white matter bundles which synapse from ocular motor control neurons (located in the brainstem) throughout the brain. These tracts may prove vulnerable to neurophysiologic changes after both phases of mTBI injury, but there remains a lack of knowledge of precisely how ocular motor deficits occur following injury. Importantly, ocular motor abnormalities appear most pronounced in tasks requiring a cognitive load, suggesting higher cortical impairment (e.g., attention, cognition, and executive function) (Balaban et al., 2016; Kelly et al., 2019; Stubbs et al., 2019; Webb et al., 2018; Wetzel et al., 2018). While advanced MRI demonstrates sensitivity to detect mTBI‐related neurophysiological and structural dysfunction, issues with the accessibility and affordability may limit widespread clinical utility of this technology. Advances in eye tracking technology may be able to overcome these barriers and provide clinicians with a portable, affordable, and objective tool to assist the clinical management of mTBI. This rapidly growing area warrants a critical approach to elucidate how functional deficits in eye movements measured using eye tracking may be explained by alterations in structural (as measured through DTI) and/or functional connectivity (as measured through fMRI) within regions of interest related to ocular motor control.

The purpose of this narrative scoping review is to collate the available MRI (specifically dMRI and fMRI) and eye tracking evidence, in addition to identifying gaps in knowledge that require attention in future work. The review is presented in three sections. Section 1 explores the anatomical control of eye movements to aid the reader with interpreting the discussion in subsequent sections. Section 2 examines the relationship between abnormal MRI findings and eye movements after mTBI based on the available evidence. Finally, Section 3 communicates gaps in our knowledge about MRI and eye tracking measures, which should be addressed in order to substantiate this emerging field.

### 1.1 Section 1: Anatomical control of eye movements

#### 1.1.1 White matter tracts in mTBI

The brain's cellular composition is broadly grouped into gray matter and white matter. Gray matter holds interneurons (confined within gray matter) and long‐projection neurons, which signal through myelinated axons to distant brain regions. These form bundles of fibers as they exit gray matter which are white in appearance (hence the term, “white matter”) (Mandonnet et al., 2018). These long‐range pathways connect neural networks. A complete description of specific white matter tracts involved in ocular motor control does not exist. Difficulties lie in correlating their structure to functional outputs as eye movement dysfunction is often attributed to multiple areas of damage (Maruta, Palacios, et al., 2016; Maruta, Spielman, et al., 2016; Maruta et al., 2010; Taghdiri et al., 2018; Ting et al., 2016).

**FIGURE 5:**
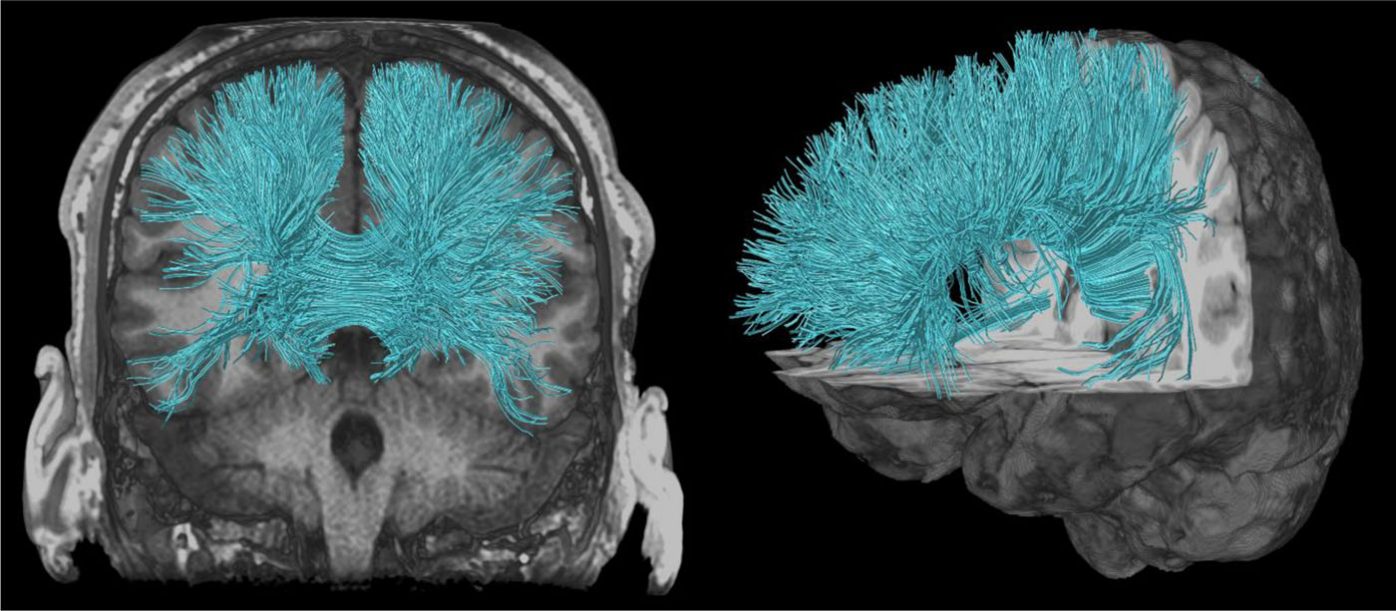
Corpus callosum (CC) illustrated with deterministic tractography with cut‐off value of 0.15 as a tensor FA threshold for terminating tracts. Twenty thousand streamlines (tracts) are generated for each bundle of tracts. Scanning was acquired on a 3‐Tesla MRI (GE Signa™ Premier) with a 48‐channel head coil and 54 diffusion gradients. Voxel size = 2 mm isotropic with three b‐values = 1000, 2000, and 3000 s/mm^2^, 15, 15, and 20 directions respectively, and 4× b = 0

**FIGURE 6:**
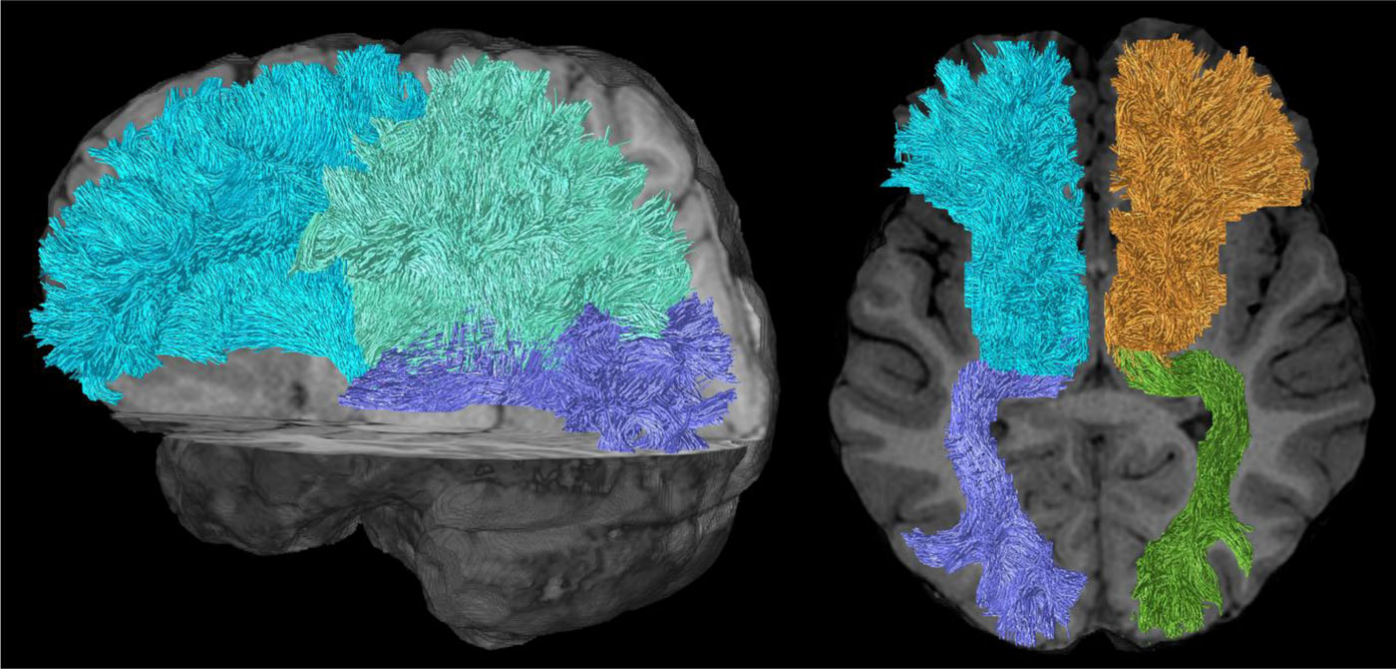
Anterior, superior, and posterior thalamic radiations illustrated with deterministic tractography with cut‐off value of 0.15 as a tensor FA threshold for terminating tracts. Twenty thousand streamlines (tracts) are generated for each bundle of tracts. Scanning was acquired on a 3‐Tesla MRI (GE Signa™ Premier) with a 48‐channel head coil and 54 diffusion gradients. Voxel size = 2 mm isotropic with three b‐values = 1000, 2000, and 3000 s/mm^2^, 15, 15, and 20 directions respectively, and 4× b = 0

**FIGURE 7:**
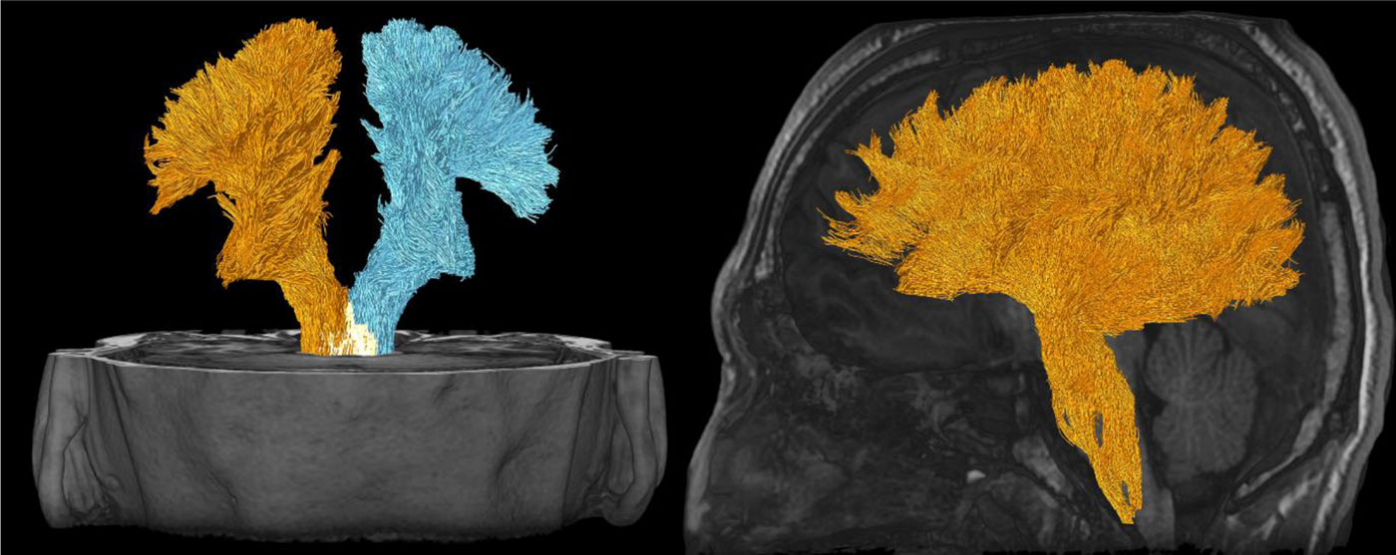
Corona radiata illustrated with deterministic tractography with a cut‐off value of 0.15 as a tensor FA threshold for terminating tracts. Twenty thousand streamlines (tracts) are generated for each bundle of tracts. Scanning was acquired on a 3‐Tesla MRI (GE Signa™ Premier) with a 48‐channel head coil and 54 diffusion gradients. Voxel size = 2 mm isotropic with three b‐values = 1000, 2000, and 3000 s/mm^2^, 15, 15, and 20 directions respectively, and 4× b = 0

**FIGURE 8:**
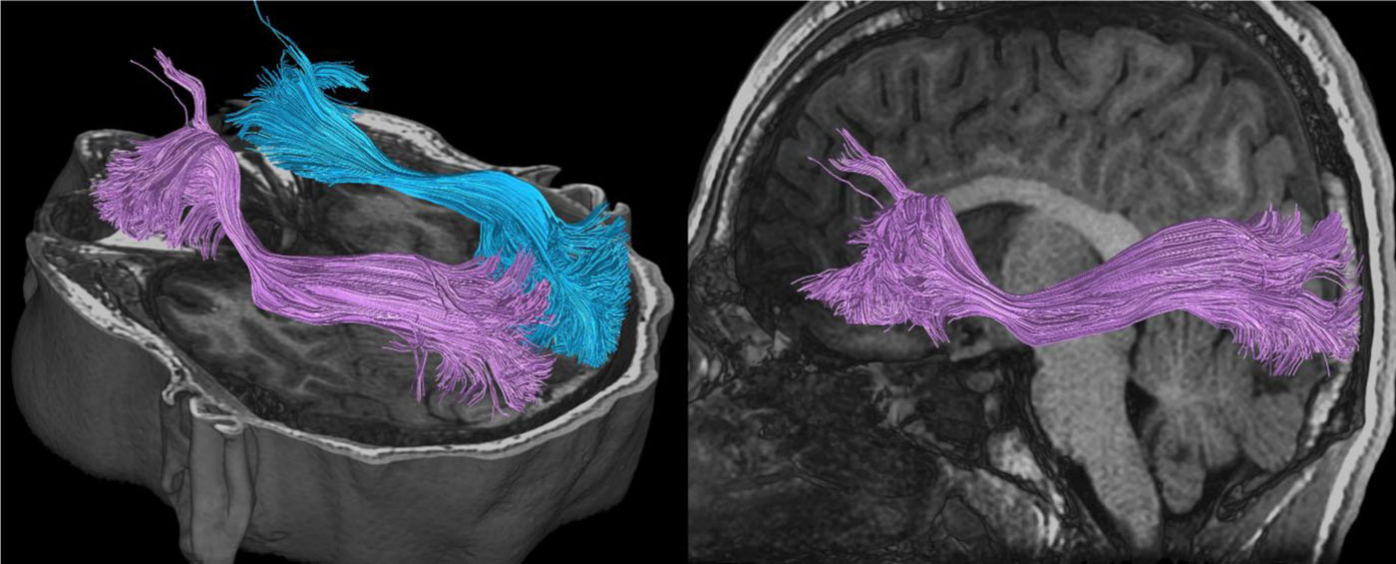
Inferior fronto‐occipital fasciculus (IFOF) illustrated with deterministic tractography with a cut‐off value of 0.15 as a tensor FA threshold for terminating tracts. Twenty thousand streamlines (tracts) are generated for each bundle of tracts. Scanning was acquired on a 3‐Tesla MRI (GE Signa™ Premier) with a 48‐channel head coil and 54 diffusion gradients. Voxel size = 2 mm isotropic with three b‐values = 1000, 2000, and 3000 s/mm^2^, 15, 15, and 20 directions respectively, and 4× b = 0

**FIGURE 9:**
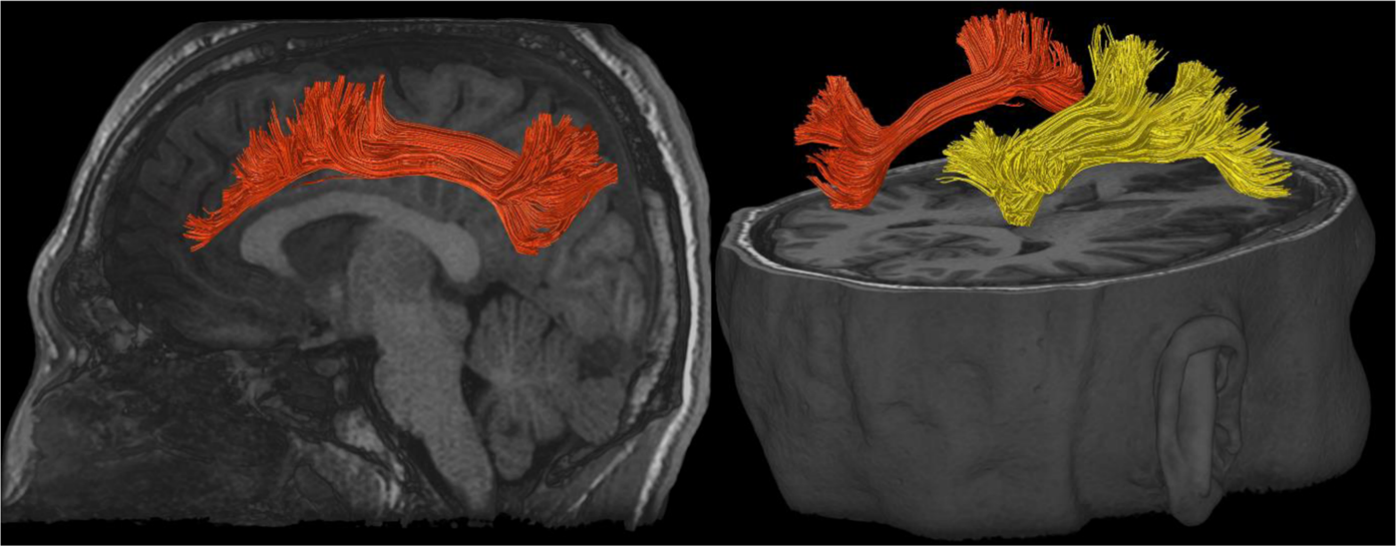
Superior longitudinal fasciculus (SLF) illustrated with deterministic tractography with a cut‐off value of 0.15 as a tensor FA threshold for terminating tracts. Twenty thousand streamlines (tracts) are generated for each bundle of tracts. Scanning was acquired on a 3‐Tesla MRI (GE Signa™ Premier) with a 48‐channel head coil and 54 diffusion gradients. Voxel size = 2 mm isotropic with three b‐values = 1000, 2000, and 3000 s/mm^2^, 15, 15, and 20 directions respectively, and 4× b = 0

**FIGURE 10:**
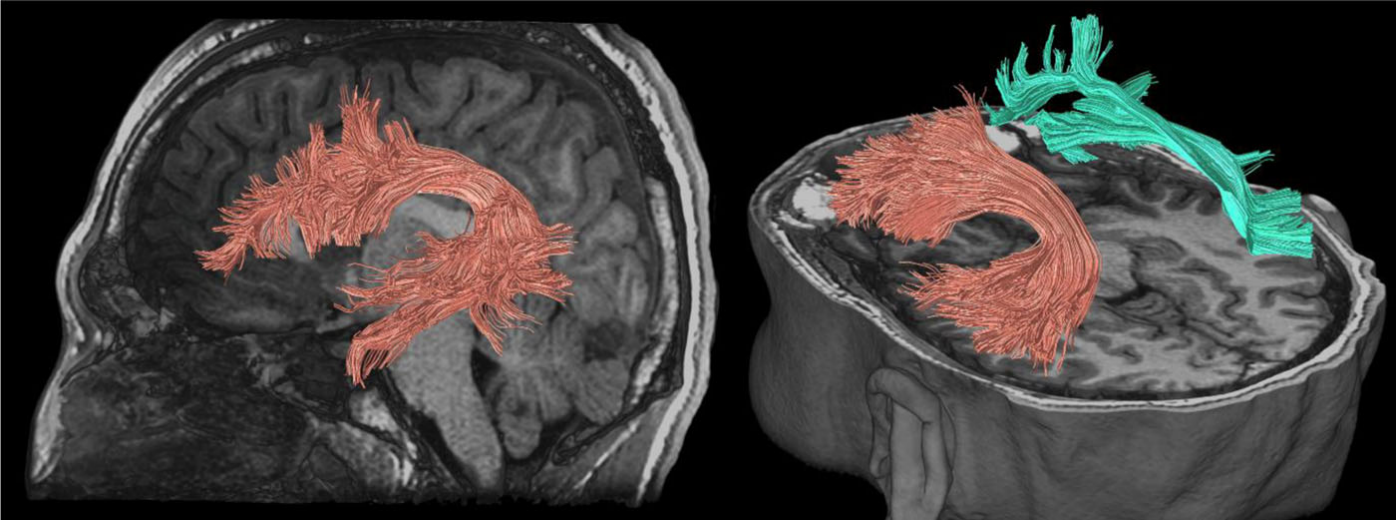
Arcuate fasciculus (AF) illustrated with deterministic tractography with a cut‐off value of 0.15 as a tensor FA threshold for terminating tracts. Twenty thousand streamlines (tracts) are generated for each bundle of tracts. Scanning was acquired on a 3‐Tesla MRI (GE Signa™ Premier) with a 48‐channel head coil and 54 diffusion gradients. Voxel size = 2 mm isotropic with three b‐values = 1000, 2000, and 3000 s/mm^2^, 15, 15, and 20 directions respectively, and 4× b = 0

**FIGURE 11:**
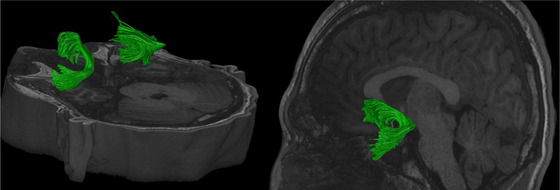
Uncinate fasciculus (UF) illustrated with deterministic tractography with a cut‐off value of 0.15 as a tensor FA threshold for terminating tracts. Twenty thousand streamlines (tracts) are generated for each bundle of tracts. Scanning was acquired on a 3‐Tesla MRI (GE Signa™ Premier) with a 48‐channel head coil and 54 diffusion gradients. Voxel size = 2 mm isotropic with three b‐values = 1000, 2000, and 3000 s/mm^2^, 15, 15, and 20 directions respectively, and 4× b = 0

**FIGURE 12:**
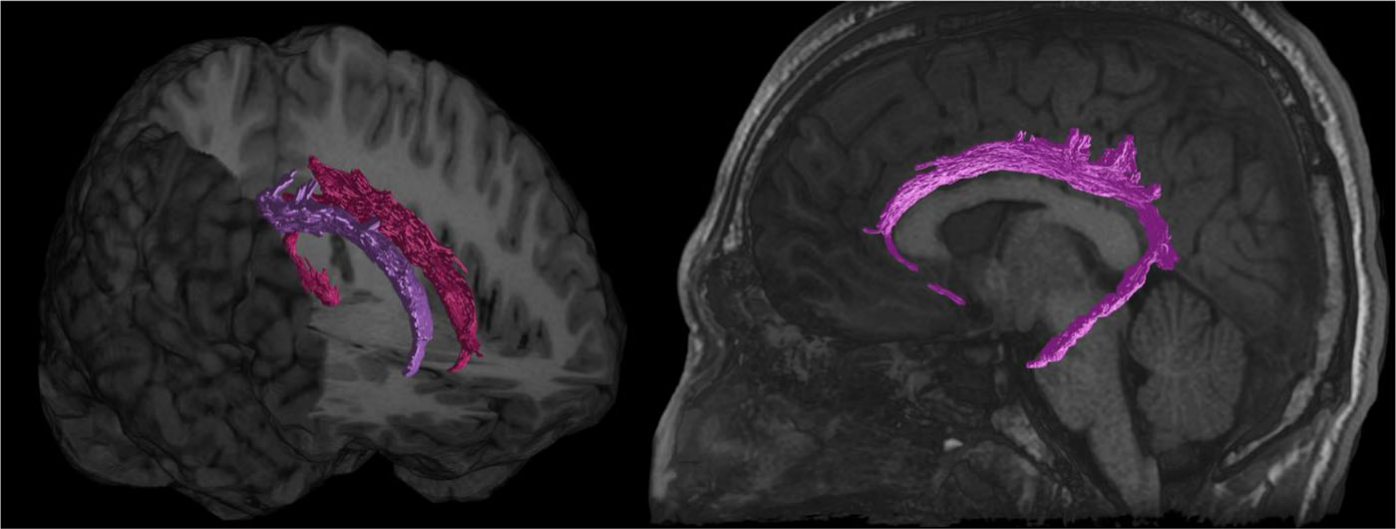
Cingulum bundle (CB) illustrated with deterministic tractography with a cut‐off value of 0.15 as a tensor FA threshold for terminating tracts. Twenty thousand streamlines (tracts) are generated for each bundle of tracts. Scanning was acquired on a 3‐Tesla MRI (GE Signa™ Premier) with a 48‐channel head coil and 54 diffusion gradients. Voxel size = 2 mm isotropic with three b‐values = 1000, 2000, and 3000 s/mm^2^, 15, 15, and 20 directions respectively, and 4× b = 0

Major white matter bundles of interest include the superior longitudinal fasciculus (SLF), corticospinal, optic radiation, corpus callosum (CC), inferior longitudinal fasciculus (ILF), inferior fronto‐occipital fasciculus (IFOF), superior fronto‐occipital fasciculus (SFOF, although existence is debated; Bao et al., 2017), sagittal stratum, arcuate fasciculus (AF), uncinate fasciculus (UF), posterior thalamic radiation, cingulate bundles (CB), and somatosensory tracts (Maller et al., 2015). Biomechanical modelling with finite element analysis based on head impact telemetry has revealed key regions at highest risk from blunt trauma due to shear stress: hippocampal (and parahippocampal), CC, midbrain, thalamus (and hypothalamus), fornix, and orbito‐frontal‐temporal regions. Deep midline structures are at a particularly high risk (Viano et al., 2005). Zhao et al. (2017) further analyzed biomechanical strain susceptibility of white matter bundles, suggesting the SLF is at the highest risk, followed by the CC, cerebral peduncle, and UF. A systematic review of diffusion tensor imaging in sport‐related mTBI revealed the following regions most frequently detected as abnormal by researchers this area: CC, internal capsule, thalamic radiations, anterior corona radiata, SLF/ILF, and the IFOF (Tayebi et al., 2021). A combination of susceptibility to biomechanical forces and frequency of citation in the literature will form the basis for the regions/tracts detailed in Table 1, which contains Figures 5, 6, 7, 8, 9, 10, 11, 12 illustrating the major white matter bundles.

**TABLE 1:** Major white matter bundles in mTBI

White matter tract	Anatomy	Figure
Corpus callosum (CC)	The CC is composed of approximately half a billion fibers Connects parietal cortices (and temporal lobes through the anterior commissure tracts) for interhemispheric communication and transduction of visual and ocular motor signals (Colby et al., 2005) The CC is sensitive to both coronal and particularly lateral impacts due to its relationship with the falx cerebri (a relatively stiff tissue) (Hernandez et al., 2019). The CC is the most commonly researched (and perhaps affected) white matter tract in mTBI (Arfanakis et al., 2002; Maruta et al., 2010; Tayebi et al., 2021), particularly in sports‐related repetitive impacts (Bazarian et al., 2007; Bazarian et al., 2014; Holcomb et al., 2021; Tayebi et al., 2021)	5
Thalamic radiation (anterior, posterior, superior, and inferior)	The thalamic radiation involves the anterior, posterior, superior, and inferior radiations which form the cortico‐basal ganglia‐thalamo‐cortical network (Maller et al., 2015; Thompson et al., 2020) These tracts operate in parallel to facilitate cognitive, motor, and affective commands (Maller et al., 2015; Thompson et al., 2020) Efferent motor commands from the superior colliculus synapse through the mediodorsal thalamus to the frontal eye field which internally monitors saccade accuracy (amplitude) (Sommer & Wurtz, 2004). The ventrolateral thalamus moderates smooth pursuit velocity and direction through projections to both the frontal eye field and supplementary eye field (Tanaka, 2005). Posteriorly, it receives subcortical input from the vestibular nuclei and deep cerebellar nuclei for the control of eye movement (Asanuma et al., 1983; Lynch et al., 1994) In mTBI, these widespread bundles are commonly affected (Cubon et al., 2011; Grossman et al., 2013; Little et al., 2010; Messé et al., 2011), which may explain feelings of headache, memory problems, insomnia, fatigue, and altered cognition in mTBI (Grossman & Inglese, 2016)	6
Corona radiata	These radiating fibres project posteriorly and converge in the internal capsule above the superior border of the lentiform nucleus. From here, they continue past the basal ganglia and terminate at the thalamus and brainstem nuclei (Emos & Agarwal, 2021) Anteriorly, this bundle synapses with the internal capsule, facilitating emotion processing, cognition, decision making, and motivation (Safadi et al., 2018) Posteriorly they merge with fibers of the posterior thalamic radiation, corticospinal tract, corticorubral tract, and corticopontine tract which are implicated in the primary motor cortex and premotor areas (Emos & Agarwal, 2021) Well reported in mTBI literature (Holcomb et al., 2021; Kasahara et al., 2012; Mayer et al., 2010; Niogi et al., 2008) and thought to be particularly susceptible to axonal injury due to its configuration (Holcomb et al., 2021).	7
Inferior fronto‐occipital fasciculus (IFOF)	An association tract connecting the frontal cortex to the posterior occipital lobe, in addition to the temporal and parietal cortices (Hau et al., 2016) Responsible for intrahemispheric relaying of information Implicated in language semantics and visual recognition (Duffau, 2015) Commonly found to show disruption in both mTBI (Jia et al., 2021; Lima Santos et al., 2021) and subconcussive impacts (Bahrami et al., 2016) due to its lengthy course from anterior/posterior, residing along a sagittal plane (Zhao et al., 2017)	8
Superior longitudinal fasciculus (SLF)	Lies above the arcuate fasciculus and is a parieto‐frontal tract, connecting the anterior cingulate cortex, the medial aspect of the superior frontal gyrus, the presupplementary motor area (pre‐SMA) and SMA, paracentral lobule, and the precuneus (Komaitis et al., 2020) Main role is visuospatial attention (Stanford Medicine, 2021), whereas the ILF connects the occipital and temporal lobes to facilitate visual processing (object and facial recognition) in addition to language comprehension (Shin et al., 2019) As one of the most biomechanically vulnerable tracts in mTBI (Holcomb et al., 2021; Zhao et al., 2017), the SLF has been frequently associated with abnormal diffusion in mTBI (MacDonald et al., 2018; Murdaugh et al., 2018), including subconcussive impacts (Bahrami et al., 2016; Sollmann et al., 2018).	9
Arcuate fasciculus (AF)	An association tract connecting the lateral temporal cortex with the frontal cortex through a dorsal pathway around the Sylvian fissure (aptly named for its “arc”‐like shape) Follows the path of the SLF but extends more temporally than this neighboring tract Favors the left hemisphere with marked asymmetry which is thought to originate from the evolution of language processing (Fernández‐Miranda et al., 2015) Terminates in the inferior frontal gyrus, ventral precentral gyrus (posterior frontal lobe), and caudal middle frontal gyrus anteriorly (Fernández‐Miranda et al., 2015) Inferiorly and posteriorly, it ends in areas of the temporal cortex responsible for object naming (Nakamura et al., 2002), recognition memory (Nakamura et al., 2002), auditory association (Wernicke's area), visual association, and attention (Patel et al., 2021) The AF's course along the Sylvian fissure, along with densely packed fiber bundles (including crossings), make this tract susceptible to rotational forces in mTBI (Cubon et al., 2011) and blast‐related forces (S. H. Jang et al., 2016)	10
Uncinate fasciculus (UF)	Arises in the orbito‐frontal cortex ventrolateral to the IFOF to connect this area to the anterior prefrontal cortex, middle frontal gyrus, and superior/middle/inferior temporal gyri (Hau et al., 2016) Its precise function is not well understood, but based on its anatomical path, it is considered to be responsible for object perception, memory, social, and emotional concepts (Von Der Heide et al., 2013) Due to its arc around the Sylvian fissure, this tract is expected to be prone to shear stress and biomechanical forces experiences in mTBI (Zhao et al., 2017) Numerous groups have correlated UF integrity to behavioral outcomes (C. P. Johnson et al., 2011), eye movement deficits (Maruta et al., 2010; Taghdiri et al., 2018), and mTBI diagnosis (Lima Santos et al., 2021; Murdaugh et al., 2018; Seo et al., 2012)	11
Cingulum bundle (CB)	Originates below the rostrum of the CC (antero‐inferior aspect) and spans across the outer borders of the CC to lie along the medial aspect of each hemisphere, superior to the CC Composed of both long and short white matter tracts, it extends laterally (forming the isthmus of the CB), anteriorly to the anterior thalamic nuclei, and ends at the hippocampal gyrus in each medial temporal lobe as well as posteriorly in the parietal cortex (Bubb et al., 2018; Stanford Medicine, 2021) Multiple groups have shown damage to the cingulum bundle in mTBI (Davenport et al., 2012; Levine et al., 2008), and subconcussive impacts (Barber Foss et al., 2019; Holcomb et al., 2021; I. Jang et al., 2019), resulting in reduced self‐paced saccades (Taghdiri et al., 2018)	12

Abbreviation: mTBI, mild traumatic brain injury.

#### 1.1.2 Gray matter control of eye movement

Direct visualization of gray matter activation during eye movement tasks has been made possible in recent history due to fMRI, highlighting the interplay between cognitive and sensorimotor brain systems. Key areas of higher cortical control of eye movements in both smoothly tracking objects and shifting gaze to new targets involve the frontal eye field, supplementary eye field (medial superior frontal cortex), precuneus, posterior parietal cortex (parietal eye field and supramarginal gyrus), junction of occipital and temporal cortex (medial temporal/ medial superior temporal cortex), and the cerebellum (Petit & Haxby, 1999; Sweeney et al., 2007). Anticipatory control recruits the prefrontal, presupplementary motor, anterior cingulate, hippocampus, thalamus, striatum, and cerebellar regions, whereas reflexive, visually guided saccades consistently activate the cortical eye fields and occipital cortex to a greater degree (Simó et al., 2005). When more complex processes are involved which require cognition, such as memory‐guided saccades (recruiting spatial working memory) or antisaccades (inhibiting a reflexive saccade to consciously gaze in the opposite direction of a target), the frontostriatal loop (dorsolateral prefrontal cortex‐DLPFC, caudate nucleus, and thalamus) is activated (Sweeney et al., 2007). Ettinger et al. (2008) evaluated antisaccades with fMRI (17 healthy volunteers), showing activation during inhibition of saccades in the right supramarginal gyrus, while the right lateral frontal eye field and bilateral intraparietal sulci generated antisaccades. Ventrolateral and dorsolateral prefrontal cortices were activated throughout which was considered to oversee the control of eye movements. Likewise, Thakkar et al. (2016) showed speed of saccade execution and inhibition to be a right‐lateralized network of frontostriatal regions. White matter tract integrity (measured via diffusion MRI) showed that connections between the FEF and SEF were correlated to faster saccade performance. Connections between the dorsal striatum and both the SEF and inferior frontal cortex (in addition to between SEF and inferior frontal cortex) correlated the speed of inhibition. A study involving isolated ischemic lesions to the FEF and parietal eye field highlighted the importance of these regions with delayed (increased saccade reaction time, known as latency) and hypometric (eye movements falling short of the target) saccades which recovered in 4 weeks after networks rearranged from physiological repair mechanisms (Nyffeler et al., 2011).

These task‐specific networks exemplify the interaction between cognition and sensorimotor control, suggesting widespread activation during ocular motor tasks. Although researchers are aware of such activation during these tasks from fMRI studies, we are yet to understand the organization of such structures (i.e., location of information gathering, order and hierarchy of networks, and white matter tract organization within areas of fMRI activation), in addition to compensatory responses when damaged. It may be more important to consider the entire brain's network disruption following mTBI, rather than focusing on one particular area or task. Early altered whole‐brain functional connectivity may result in a global delay in information processing, cognition, and executive function contributing to ocular motor dysfunction (Shumskaya et al., 2012).

#### 1.1.3 Efferent control of eye movement: Cranial nerves III, IV, and VI

**FIGURE 13:**
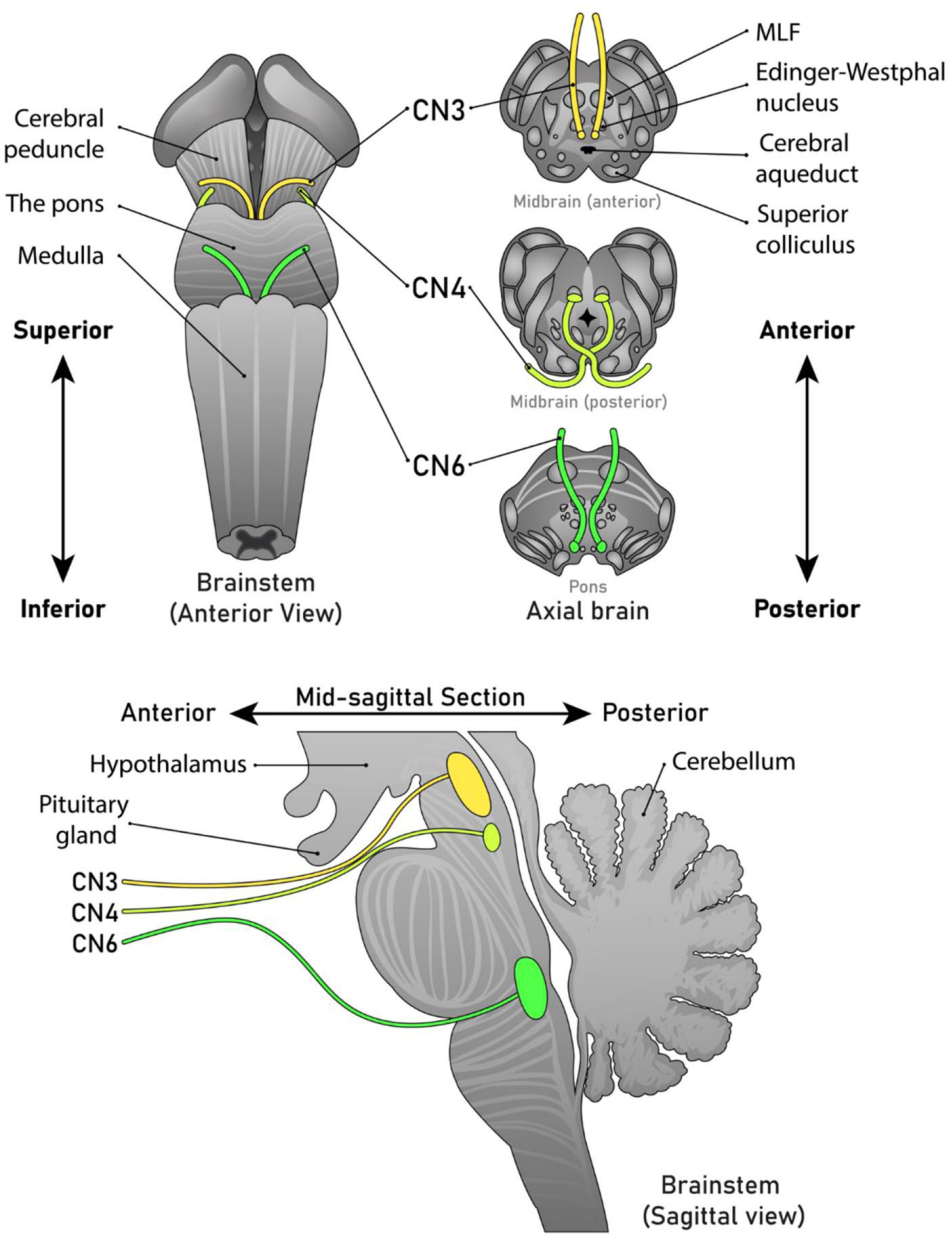
Anterior, axial, and sagittal views of the anatomical origins of cranial nerves III, IV, and VI

Efferent control of eye movement involves the integration of cranial nerves (CN) III, IV, and VI (detailed in Table 2 with anatomical origins in Figure 13). Cranial nerves III, IV, and VI are influenced by visual perception, processing (higher cortical input), and cranial nerve output which results in gaze. White matter tracts (Table 1) serve these areas, allowing for interneuronal communication. The frontal eye field (FEF) and supplementary eye field (SEF) are brain regions which innervate the paramedian pontine reticular formation (PPRF) for lateral gaze (Sakai et al., 2014). The PPRF is ventral to the CN VI nuclei and the MLF which is midline in the pons and forms the lateral gaze center. Convergence and divergence signals are generated by convergence and divergence cells within the superior colliculus, which signal independently to the abducens internuclear neurons (Mays, 1984; Van Horn et al., 2013). Vertical gaze is controlled at the rostral interstitial nucleus of the MLF, which resides near the CN III nucleus (midbrain), in addition to the Cajal interstitial nucleus (Sakai et al., 2014). Conjugacy (ability of the eyes to work in unison for binocular vision) results from connections of fibers through the MLF which is continuous with the spinal cord and contains descending fibers to govern head position and posture (Sakai et al., 2014). Altogether, these pathways result in the orchestration of ocular motor control.

**TABLE 2:** Efferent control of eye movement

Cranial nerve	Anatomy
Oculomotor (III)	CN III nuclei are located in the midbrain in the parasagittal ventral apex around the aqueduct, adjacent to the medial longitudinal fasciculus (MLF) and superior colliculi (SC) Contain a portion of fiber tracts that travel anteroposteriorly on tractography through the tegmentum In the midbrain, CN III synapses across the parasagittal midline, exiting at the interpeduncular fossa to the cavernous sinus (Sakai et al., 2014) CN III contains accessory nuclei (e.g., Edinger‐Westphal nucleus), which form an efferent pathway to the medial, superior, and inferior rectus, in addition to the inferior oblique and levator palpebrae (eye lid control) Its parasympathetic pathway innervates the ciliary ganglion for control of the ciliary muscle and sphincter pupillae (pupillary response)
Trochlear (IV)	Nuclei are caudal to the oculomotor nuclei in the midbrain Fiber tracts exit the brainstem to travel along the ambient cistern (posterior to thalami), traveling around the periaqueductal gray matter to decussate at the superior medullary velum, between the cerebral peduncles (Sakai et al., 2014) From the cerebral peduncles tracts lead anteriorly along the middle cranial fossa to the outer wall of the cavernous sinus to the superior orbital fissure to innervate the superior oblique muscles (Rea, 2014a)
Abducens (VI)	Nuclei reside in the mid‐lower portion of the pons near the facial colliculi (facial nerve, CN VII) Travels inferiorly through the medial leminiscus (a major tract responsible for proprioceptive input, synapsing with the thalamus) and the corticospinal tract (Sakai et al., 2014) Fibers exit the brain stem through the pontomedullary groove and prepontine cistern, traveling dorsal to the anterior inferior cerebellar artery into the dura mater, crossing the inferior petrosal sinus (Rea, 2014b) This long intradural course leads into the abducens foramen (anchored inside Dorello's canal) to the cavernous sinus From the cavernous sinus, it enters the superior orbital fissure to innervate the lateral recti

### 1.2 Section 2: Relationship between MRI and eye movements after mTBI

The most promising method of unraveling the pathophysiology behind ocular motor impairment following mTBI is in vivo correlation using structural (dMRI) and functional (fMRI) imaging. Although studies have started to employ advanced MRI methods on mTBI patients with ocular motor dysfunction, previously these areas have been studied in isolation, questioning the pathophysiological basis of eye movement dysfunction. Although the literature remains limited, there are seven studies which focus on dMRI and eight studies for fMRI correlates. Only two studies have used multimodal imaging (both dMRI and fMRI) alongside eye tracking indices (Astafiev et al., 2015; Clough et al., 2018). “Chronic mTBI” (> 2 months postinjury with ongoing symptom burden) form the overwhelming majority of subjects in these studies, likely due to ease of recruitment through concussion clinics, rather than emergency departments where only a minority present (Sye et al., 2006; Zhang et al., 2016). Eye tracking experiments include smooth pursuit (Maruta, Palacios, et al., 2016; Maruta, Spielman, et al., 2016; Maruta et al., 2010) (Johnson, Hallett, et al., 2015; Johnson, Zhang, et al., 2015), self‐paced saccades (Taghdiri et al., 2018), reflexive saccades (Clough et al., 2018) (Johnson, Hallett, et al., 2015; Johnson, Zhang, et al., 2015) (Symons et al., 2021), fixation (Johnson, Hallett, et al., 2015; Johnson, Zhang, et al., 2015), memory‐guided (Johnson, Hallett, et al., 2015; Johnson, Zhang, et al., 2015), reading (King‐Devick test) (Kaushal et al., 2019), and antisaccades (Ting et al., 2016) (Clough et al., 2018; Johnson, Hallett, et al., 2015; Johnson, Zhang, et al., 2015) (Symons et al., 2021).

#### 1.2.1 Diffusion MRI correlates with eye movements in mTBI

Diffusion characteristics of white matter tracts are key surrogates for axonal injury where trauma‐induced swelling and stretching of axons breaks neuronal connections (Blumbergs et al., 1994; Budde et al., 2011). To understand dMRI outcomes measures, it is helpful to visualize the diffusion tensor as a spherical or ellipsoid shape (Figure 3). From the diffusion tensor, four measures are typically calculated:

(1) Fractional anisotropy (FA). This is particularly useful for visualization of white matter tracts which contain myelinated nerves with few cell bodies (longer range transmission), in comparison to gray matter containing cell bodies with less myelinated nerve tissue (shorter range transmission) (Suri & Lipton, 2018). Low FA will alter the diffusion ellipsoid to a sphere (minimum FA value of 0), whereas increasing anisotropy will elongate the tensor to an ellipsoid (maximum FA value of 1) (O'Donnell & Westin, 2011). In the context of mTBI, decreased FA may be due to injury to a white matter tract.
(2) Mean diffusivity (MD), the “size” of the ellipsoid rather than the “shape,” measures the diffusion magnitude (i.e., average diffusion of water molecules within a voxel), which may also be disrupted following injury.
(3) Axial diffusivity (AD) and radial diffusivity (RD) represent the average of water diffusion running parallel (AD) and perpendicular (RD) to the principal axis of direction, or white matter tract (Winklewski et al., 2018).

Within the last decade, groups have begun to evaluate white matter tracts using diffusion MRI to correlate eye movement dysfunction in mTBI patients (Maruta, Palacios, et al., 2016; Maruta, Spielman, et al., 2016; Maruta et al., 2010; Taghdiri et al., 2018; Ting et al., 2016). However, only one group focuses on acute cohorts (Ting et al., 2016). Many of these studies cite various diffusion parameters, from 55‐direction HARDI (b = 1000 s/mm^2^) (Maruta et al., 2010), 60‐direction diffusion gradients (b = 1000 s/mm^2^) (Taghdiri et al., 2018), 12‐directions (b = 1000 s/mm^2^) (Ting et al., 2016), and 64‐directions at (b = 3000 s/mm^2^) (Clough et al., 2018). This is important to consider for the generalizability and scientific validity of these results. Of the few studies available exploring ocular motor abnormalities in dMRI, studies are categorized via acute (< 1 month postinjury) and chronic (> 2 months postinjury) below.

##### Acute mTBI cohorts

Ting et al. (2016) evaluated antisaccade measures in acute mTBI patients (11 patients, < 1 week postinjury with follow up between 2 and 4 weeks postinjury), chronic mTBI patients (15 patients with ongoing symptom burden > 3 months postinjury; data collection at one time point), and 10 healthy controls (eye tracking was performed twice, 2–4 weeks apart, with one MRI scan). The majority of the acute mTBI patients (*n* = 9) had their single MRI scan at the follow‐up appointment. In this cohort, MD was increased in the splenium of the CC which positively correlated to increased latency of antisaccades. This persisted to their second follow‐up at 4 weeks. Their magnitude of eye movement error was also positively correlated to symptom burden. The chronic mTBI cohort showed decreased MD in the corticospinal tract only, compared to controls, in addition to increased antisaccade latency. Interestingly, there was also a negative association between antisaccade performance and Stroop color‐word score, a measure of executive neurocognitive function.

Ting's group published a further study in 2020 which investigated how these ocular motor deficits occurred in acute mTBIs from a biomechanical perspective (8 patients, < 1 week postinjury with five controls who were age‐, gender‐ and education matched) using a similar DTI acquisition protocol of 11 noncollinear diffusion directions with b = 1000 s/mm^2^ (Post et al., 2019). The clinical neurotrauma report form with patient history was used to simulate the impact with headforms (ballistic models) and accelerometers in a biomechanical laboratory setting following injury. In this pilot study of eight patients, they noted increased saccade latency only in the mTBI group (duration, amplitude, and velocity were not significant) which was associated with a decrease in RD and AD in the cerebral peduncle and cingulum hippocampus. AD was increased in the corona radiata. Their biomechanical variables did not explain the increased latency which was considered to have been due to other factors such as “attentiveness and wakefulness,” in addition to a lower adaptive ability to higher cognitive loads following injury. A key limitation to this study is not only its sample size, but failure to investigate causes of ocular motor dysfunction from the second phase of mTBI (neurometabolic cascade) and additional gray matter injury from a functional perspective.

##### Chronic mTBI cohorts

**FIGURE 14:**
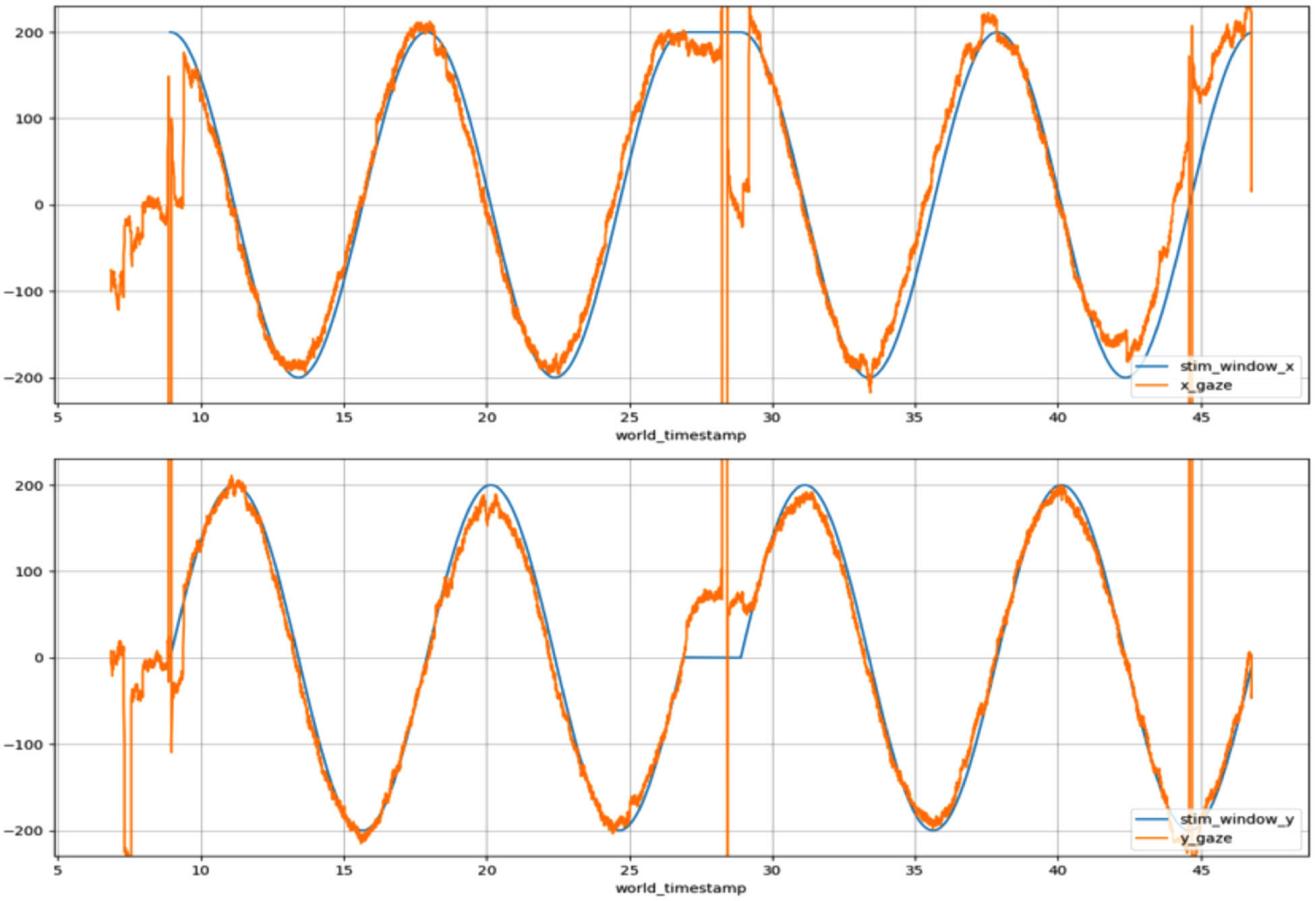
Smooth pursuit gaze trajectory (orange line) along a circular target path (blue line) of a healthy participant at 200 frames per second. Horizontal and vertical coordinates are separated above and below, respectively. High amplitude vertical orange lines represent blink events, which are typically excluded from analysis

Maruta et al. (2010) studied a cohort with persistent symptom burden (termed “postconcussion syndrome”) of 17 patients (6 weeks to 5 years postinjury; average 2.7 years) compared to nine healthy subjects. The persistent symptom burden cohort showed significantly impaired circular smooth pursuit measures that correlated to white matter changes on HARDI (55 directions) when compared to controls. Specifically, disrupted FA of the right anterior corona radiata (ACR), UF, and genu of CC led to increased radial and tangential errors in eye tracking measures. Gaze error variability was also correlated with increased FA in the forceps major and superior cerebellar peduncle. Maruta, Palacios, et al. (2016) published a follow‐up study where they compared 32 “milder” mTBI patients (less severe injuries than previous cohort as defined by absence of contusion on MRI report) with ongoing symptoms (90 days to 5 years prior to testing) to 126 control subjects. This investigation only studied FA and averaged all subjects (both case and control) into their FA map. Damaged white matter structures were defined as abnormal if the FA value was “less than the mean minus 2.5 times the standard deviation of the 126 control subjects” (p. 3). In both case‐control and group‐wise comparisons, they did not find any significant changes between eye tracking metrics or white matter tracts (measured via FA values only). However, in six of the 32 patients, they reported damage to white matter tracts, but these patients with positive DTI findings were not presented individually. This suggests that milder mTBIs may be more difficult to detect with advanced MRI and eye tracking. A second analysis was performed on the milder injury cohort where an attention‐demanding task was performed prior to eye tracking (Maruta, Spielman, et al., 2016). With a higher cognitive load, these patients demonstrated greater variability in smooth pursuit performance which was attributed to higher fatigability and lower cognitive reserves in these patients. Figure 14 demonstrates an example of a smooth pursuit eye tracking protocol.

Taghdiri et al. (2018) assessed 59 patients with postconcussion syndrome (25.9 months postinjury ± 63.6 months, mean 12 months, max 39 years). In this cohort, FA of the left UF mediated the relationship between the time of last concussive impact to the number of self‐paced saccades, while FA of the left cingulum mediated the relationship between their total symptom burden to number of self‐paced saccades as a combined outcome. This analysis focused on the bilateral tracts of the SLF, UF, cingulum, and CC, where the SLF and CC were not proven significant mediators of symptom burden or self‐paced saccades. The large variability in their patient cohort likely affected the statistical power and validity of these results.

Clough et al. (2018) investigated long‐term white matter tract changes on a 15‐patient Australian rules football player cohort (no mTBI in past 6 months, but repeated exposure to head impacts; ages 24.3 ± 0.9 years) using DTI (diffusion‐weighted gradients applied in 64 directions), fMRI, and ocular motor tasks (prosaccades and antisaccades). While they did not show any significant MRI changes between groups, they found significantly impaired “switch cost” (increased eye tracking errors when moving between prosaccades and antisaccades) with a shorter saccade latency. Their follow‐up study in 2021 increased their cohort to 26 male asymptomatic Australian rules football players (no mTBI in past 6 months, but history of multiple head impact exposure) who were also matched to age‐similar controls (23 noncollision athletes) (Symons et al., 2021). Antisaccade latency was increased in footballers with mTBI history, along with impaired switch cost, similar to their previous study. With greater statistical power, their DTI analysis showed decreased FA in the CC and corticospinal tract relative to controls. Their prosaccade “switch cost” correlated to reduced FA of anterior white matter regions connecting the prefrontal cortex (i.e., DLPFC) which is known to be involved with executive functioning and task switching. Altogether, their results support the notion that long‐term, repetitive damage to cognition/attention‐related ocular motor structures lead to readily detectable ocular motor abnormalities in more complex saccade tasks.

In summary, there is limited literature correlating ocular motor dysfunction to white matter tract abnormalities in mTBI. However, when eye movements were evaluated, abnormalities were most pronounced in those with a higher symptom burden (e.g., increased antisaccade latency in Post et al. (2019) and Ting et al. (2016)). Commonly disrupted white matter tracts included the ACR (Maruta et al., 2010), UF (Maruta et al., 2010; Taghdiri et al., 2018), cingulum (Post et al., 2019; Taghdiri et al., 2018), genu of CC (Manning et al., 2020; Maruta et al., 2010; Ting et al., 2016), and cerebellar peduncle (Maruta et al., 2010; Post et al., 2019). In general, heterogeneity in methodology (MRI scanners, regions of interest selected to analyze, differences in participants and mechanism of injury, comparison groups, sample size, and confounding variables such as repeat mTBI victims with both old and new lesions) makes it difficult to draw uniform conclusions in dMRI, even in larger studies (Tayebi et al., 2021). However, acquisition parameters and analysis methods continue to improve. Overall, the limited evidence available from mostly chronic mTBI cohorts show white matter tract abnormalities in addition to ocular motor dysfunction, but whether they are biologically connected remains to be substantiated by additional fMRI/ multimodal analysis.

### 1.3 Functional MRI correlates with eye movements in mTBI

An extensive body of literature in brain injury has used fMRI to show disruption to key neural pathways during brain injury, particularly in rs‐fMRI networks. This includes the right fronto‐parietal network (i.e., DLPFC region) (Czerniak et al., 2015; Mayer et al., 2011; Shumskaya et al., 2012; Slobounov et al., 2011; Sours et al., 2015) and visual networks (Manning et al., 2020). The default mode network (DMN) (Figure 4) is the brain's baseline state which shows activation when one lies quietly awake, with eyes open or closed (performed during a typical rs‐fMRI) (Raichle et al., 2001). However, this is less useful than studies who have performed task‐based fMRI when the aim is to observe gray matter behavior *during* ocular motor dysfunction in those with mTBI.

Once fMRI data is collected, there are numerous forms of analysis (which exceeds the scope of this review) depending on task‐based or resting‐state data. These methods, which are an active area of research with no global consensus, share a common theme in understanding nodes and networks to derive meaningful conclusions (Stanley et al., 2013). Researchers are limited by voxel size in defining neural connections and it is not possible, nor clinically meaningful, to map billions of neurons through their thousands of individual synapses. Functional connectivity (FC) analysis is also limited by statistical dependencies without any consideration of neural network structure or system function. FC provides evidence toward correlations, but no further insight to answer the question: are the two areas of activation in a time series correlated because “(1) x influences y, (2) y influences x, (3) both influence each other, or (4) both are influenced by a third variable?” (Stephan & Friston, 2009)(p. 391).

Select groups have investigated whether eye tracking deficits in mTBI patients correlate to fMRI metrics (Clough et al., 2018; Johnson, Hallett, et al., 2015; Johnson, Zhang, et al., 2015; Kellar et al., 2018; Rockswold et al., 2019). Of the limited literature available exploring ocular motor dysfunction in fMRI, studies are categorized via acute (< 1‐month postinjury) and chronic (> 2‐months postinjury) cohorts. Within these sections, methods (rs‐fMRI and task‐based fMRI) are described to organize the interpretation of currently available evidence.

#### 1.3.1 Acute mTBI cohorts

Johnson, Zhang, et al. (2015) evaluated saccades and smooth pursuit in nine patients (aged 18‐21) who experienced a sport‐related mTBI within 7 days of task‐based fMRI (compared to nine control participants aged 20–22). They showed an increased latency time in the saccadic tasks, increased magnitude of position errors, and less numbers of self‐paced saccades compared to their control group. Notably, their eye tracker only operated at 60 Hz, and this will have been further affected by a three‐point averaging filter used to decrease noise, degrading the precision of their measures. Their fMRI data showed “widespread increased activation of multiple brain areas…regardless of task difficulty” (p. 569). Specifically, increased BOLD signal occurred in the cerebellum, visual cortices, DLPFC, anterior cingulate cortex (ACC), and mid‐frontal gyrus for both antisaccadic tasks and self‐paced saccades. Memory‐guided saccades showed hyperactivation within the hippocampi, cerebellar tonsils, and precuneus compared to controls. A previous fMRI study also showed disruption in the hippocampi and ACC in traumatic axonal injury following mTBI which supports this finding (Marquez de la Plata et al., 2011). Johnson, Hallett, et al.’s (2015) follow‐up study showed improvement at 30 days with patients clinically asymptomatic, although hyperconnectivity remained with similar findings. This supports the hypothesis of a compensatory neural mechanism following injury which recruits additional areas for a given task. More demanding ocular motor tasks tests the functional limit of these additional neural reserves. When patients are asymptomatic and cleared for return to sport, these proposed pathophysiological signs were shown to remain at 30 days (Johnson, Hallett, et al., 2015).

Recently, Kaushal et al. (2019) performed a prospective rs‐fMRI study using a whole‐brain connectome approach in 62 sport‐related mTBIs (scanned in the acute, within 48 hours, and subacute, 8–45 days, phase of injury) which were age‐ and demographic matched to 60 controls. Although their eye movements were not tracked quantitatively, they performed the King‐Devick test which is a saccade‐based task, integrating attention and language as the patient reads out rows of spaced numbers (not performed in the scanner and only final score on this test was reported with no indication of time to complete) (Galetta et al., 2011). The King‐Devick test was also correlated to symptom scores and neurocognitive evaluation which were all found to be impaired relative to control at 8 days postinjury (no between‐group differences were found at the 48‐h, 15‐day, or 45‐day visits). Their group quantified rs‐fMRI abnormalities by nodal strength, a form of network‐based statistics where the sum of weights of all the edges of an activated region were measured. This revealed abnormalities at 8 days postinjury in the mTBI cohort which was only present in those with ongoing symptoms. There was no significant difference across asymptomatic injured and controls, explaining their group effect at this time point. Beyond 8 days, this returned to normal. Their finding of heightened connectivity (recruitment of other networks in compensation for injured regions) is supported by the literature (Czerniak et al., 2015; Kaushal et al., 2019; Tang et al., 2011) and was considered to occur from diffuse axonal injury in mTBI and alterations in cerebral blood flow, as mentioned above.

#### 1.3.2 Chronic mTBI cohorts

Rockswold et al. (2019) evaluated 10 mTBI subjects (mechanism of injury not described) with ocular motor dysfunction (in addition to symptoms of dizziness and loss of balance) diagnosed by an optometrist (inclusion criteria: impaired vergence, accommodation, smooth pursuit, or saccades; time since injury 89 ± 16 days) to a group of 9 mTBI patients who were not found to have clinically altered ocular motor function (comparison group; time since injury 66 ± 9 days). Their vergence‐based fMRI task revealed significantly decreased BOLD signal in the ocular motor dysfunction group in the medial occipital lobe (specifically the left posterior lingual gyrus, bilateral anterior lingual gyrus and cuneus), and the parahippocampal gyrus (medial temporal lobe), based on their preselected regions for analysis (this did not include brainstem ocular motor control neurons). Further analysis showed decreased functional correlation within the lingual/parahippocampal region, left middle frontal gyrus, and DLPFC. It is worth noting there were seven patients in the clinical ocular motor dysfunction group with a history of recurrent mTBI and only two in their comparison group of no‐clinical‐ocular‐motor‐dysfunction mTBI. This may have increased their effect seen, in addition to the ocular motor dysfunction cohort being more symptomatic. Symptom burden was not described in their comparison group. Additionally, it is not reported whether the optometrist who selected patients for ocular motor dysfunction diagnosed these through objective means, such as through eye tracking.

Tyler et al. (2015) investigated brainstem ocular motor control nuclei activation on task‐based fMRI during a saccade test and vergence cue in the scanner. The sample consisted of 12 mTBI patients (mechanism of injury not described), 2 months to 35 years postinjury (mean 2.2 years), and 11 age‐matched controls. Detailed saccade and vergence parameters were evaluated prior to their scanning session (outside of the scanner) and consisted of only 12 trials per eye (1 minute in duration with missing data due to blinks not reported), showing increased latencies (saccades), slower velocities (saccades and vergence), and larger asymmetry in the mTBI cohort. In the brainstem, this lead to a 50% reduction in activation of both the ocular motor nuclei and superior colliculi relative to controls, whereas for vergence, the supraoculomotor area showed the most dramatic reduction relative to control. There was no subgroup analysis of patients 2 months postinjury and decades later, but this group suggested these ocular motor deficits persisted beyond symptomatic recovery. These results have not been replicated and should be interpreted with caution. Their eye tracker's calibration methods, precision, and accuracy were not detailed, along with test–retest reliability of saccade measures and fMRI signals for each participant. Additionally, it is unclear as to whether these fMRI signals reflected epiphenomena such as reduced visual search, fixation, or accommodation. By having participants rely on commands inside the scanner (no eye tracking was performed in the scanner itself), there is potential for participants to not follow instructions carefully with no safeguards reported by investigators to control for this.

Astafiev et al. (2015) performed a multicenter cross‐sectional, case–control study with 45 chronic mTBI patients (3 months to 5.5 years postinjury, mixed sport and non‐sport related) which correlated smooth pursuit to BOLD signals (task‐based fMRI with smooth pursuit) and diffusion MRI (DTI; 64‐directions with b = 0 and b = 1000). No difference was found in smooth pursuit tracking dysfunction (radial/tangential error or number of saccades) between the groups, but they did note a trend for increased variability. This was considered to be due to heterogeneity in sample (injury severity and time since injury) in addition to different eye trackers. However, during the circular smooth pursuit task, control patients showed consistent activation of the dorsal attention network (FEF, posterior intraparietal sulcus, ventral intraparietal sulcus, and medial temporal complex), visual cortex, cerebellum, putamen, and thalamus. This varied significantly in the mTBI cohort, which showed significantly decreased activity in different cortical, subcortical, and white matter regions. This effect was most significant in the right inferior frontal gyrus, basal ganglia, and surrounding white matter regions. However, these abnormal BOLD signals were not considered to be associated with abnormal DTI parameters (FA, AD, RD, and MD) between the two groups, except for one significant correlation which was found between AD and BOLD magnitude in the internal capsule using a Mann‐Whitney U test. However, this did not survive further analysis using a voxel‐wise comparison (tract‐based spatial statistics, TBSS). Overall, it was considered that BOLD signal difference may have been due to neurometabolic changes following injury such as GABA signaling, proving more sensitive than their diffusion protocol (Astafiev et al., 2015).

Kellar et al. (2018) studied a preseason, non‐injured cohort of 21 American football players (19 non‐contact sport controls, and 11 non‐athletic controls) to investigate any possible long‐term, cumulative impact exposure on smooth pursuit tracking during task‐based fMRI. They did not find any significant differences in slow, medium, or fast pursuit of their target (measured via one‐way analysis of variance and effect size) but did show consistently greater activation in brain regions during the slow‐ and medium‐speed smooth pursuit task. In the faster pursuit task, the cerebellum showed greater activation than the FEF, which was not the case with control groups. They considered this difference to be from brains “working harder” (a compensation theory) from long‐term subconcussive impacts, or simply because these were top athletes with a high degree of visuo‐motor skill. A similar cohort of 15‐football player cohort who had *not* experienced an mTBI in the past 6 months was evaluated by Clough et al. (2018). However, their eye tracking was performed outside of the scanner and their fMRI studied seven resting state networks only: visual, somatomotor, dorsal attention, ventral attention, limbic, frontal‐parietal, and default mode. Their justification for this sample was to find long‐term, cumulative head impact exposure evidence radiologically. Although no between‐group differences were found on fMRI, they showed significantly increased difficulty in switching from a prosaccade to antisaccade task which was felt to represent persistent cognitive changes. This study would have been strengthened by a task‐based fMRI protocol evaluating the proposed ocular motor dysfunction.

Overall, the limited literature correlating fMRI signals to ocular motor dysfunction in mTBI do not provide a unifying hypothesis. Kaushal's largest cohort with prospective whole‐brain connectome (nodal point) analysis (rs‐fMRI data) falls short of quantitative eye tracking measurement which means subtle abnormalities in ocular motor dysfunction (e.g., fixational stability or saccadic velocities) will have been missed. In addition, any large individual variability will have been lost in group‐wise analysis. In spite of this limitation, select groups found a correlation between fMRI signals to ongoing symptom burden. However, the strength of correlation between ongoing symptoms and ocular motor dysfunction remains unclear. The lack of persistent fMRI abnormality beyond symptom resolution contradicts Johnson et al.’s smaller cohort of increased activation persisting beyond this period. This may be attributed to either differences in analysis or cohort selection. Kellar et al.’s (2018) group suggests athletes with high visuomotor skill may have different patterns of activation from either compensation from previous injury, or by virtue of being an athlete which may require sports‐related mTBI to be analyzed separately to non‐sports‐related mTBI in future. Studies with longer periods postinjury (Astafiev et al., 2015; Rockswold et al., 2019; Tyler et al. (2015)) showed decreased BOLD signals in regions of interest. Rockswold et al.’s (2019) design was unique in their study of clinical ocular motor dysfunction, but no eye tracker was used for more precise quantification of these measures. Their decreased signal during a vergence‐based task suggests differences in these patients which correlate to some areas of eye movement control (medial occipital lobe, medial temporal lobe, lingual/parahippocampal region, left middle frontal gyrus, and DLPFC) but has not been replicated thus far. Astafiev et al. (2015) also showed decreased activation in patients during a smooth pursuit in a more chronic phase of injury (most significant in the right inferior frontal gyrus and basal ganglia). This is supported by Tyler et al. who showed decreased activations of specific brain stem motor areas in their patient cohort (specifically reduced activation of both the ocular motor nuclei and superior colliculi). Altogether, there remain significant gaps in our knowledge with regard to gray matter's role in ocular motor dysfunction, but these early studies are important to inform future research in this area.

### 1.4 Section 3: Areas requiring further research

Of the studies described in this review, there are clear gaps in our knowledge surrounding the relationship between mTBI‐related pathophysiology and ocular motor dysfunction. Notable areas include acute cohorts, sex differences, methodological considerations, and mechanism of injury.

#### 1.4.1 Acute mTBI cohorts

There is a paucity of literature correlating MRI abnormalities to ocular motor dysfunction in acute mTBI cohorts. This is particularly important as eye tracking irregularities are possibly more pronounced at this stage (Balaban et al., 2016; Hoffer et al., 2017; Kelly et al., 2019) (DiCesare et al., 2017; Heitger et al., 2002; Maruta et al., 2014). There are only two studies, performed at the same institution, exploring DTI abnormalities and ocular motor dysfunction (measured as antisaccades) (Post et al., 2019; Ting et al., 2016). In fMRI literature, there were only two studies as well: Johnson, Zhang, et al.’s (2015) early study was composed of only nine patients, while Kaushal et al. (2019) did not perform quantitative eye tracking, making it difficult to draw any specific or generalizable conclusions for these patients.

#### 1.4.2 Sex differences

There are limited studies examining female populations in mTBI and nearly all studies evaluating ocular motor measures with cohorts inclusive of females did not perform a subgroup analysis or compare results between genders (Balaban et al., 2016; Cochrane et al., 2019; DiCesare et al., 2017; Heitger et al., 2004, 2006, 2007, 2008, 2009; Howell et al., 2018; Kelly et al., 2019; Maruta et al., 2013; Maruta et al., 2017; Maruta, Palacios, et al., 2016; Maruta et al., 2018; Murray et al., 2014; Webb et al., 2018; Wetzel et al., 2018). This is an important consideration, as mTBI incidence is greater in females for gender‐comparable sports (Covassin et al., 2016; Dick, 2009; Gessel et al., 2007; Lincoln et al., 2011; Marar et al., 2012). When injured, females have been found to report higher symptom scores, particularly during vestibulo‐ocular reflex (VOR) and vestibular ocular motor screening (VOMS) assessments (Sufrinko et al., 2017) with a prolonged symptom burden (Baker et al., 2016; Broshek et al., 2005; Preiss‐Farzanegan et al., 2009). One study by Hoffer et al. (2017) examined 106 mTBI patients (34 female, 72 male) across three time points (initial visit, 7–10 days, and 10–17 days). There was no significant difference between male and females for their outcomes measures: symptom assessment, head impulse testing, prosaccade error rate, and smooth pursuit velocity gain during optokinetic nystagmus testing. However, a large study in a healthy cohort (413 male, 645 female, ages 16–40) revealed that males had lower saccade (and antisaccade) latency, less dynamic overshoots, less antisaccade error rates, and less antisaccade gain (Bargary et al., 2017). For smooth pursuit, males had greater smooth pursuit gain (less hypometric than females), greater catch‐up saccades, less anticipatory saccades, and lower smooth pursuit latency. This warrantsdeserves further study if eye tracking is to be considered a future clinical tool.

MRI studies comparing males to females post‐mTBI remain scarce with select studies focusing on female cohorts only. Accordingly, one group (Chamard et al., 2016) confirmed alterations in diffusion measures in the CC of female contact‐sports players, similar to male counterparts following mTBI (Henry et al., 2011; Kinnunen et al., 2011). A small study of 18 males and 14 females (30 sex‐, age‐, and education‐matched healthy controls) found significant differences in cortical thickness within 1 week of mTBI (mixed‐cause). Specifically, females had increasedthicker cortical thickness of the left caudal ACC. It is not known, however, whether this is from chance alone, mechanism of injury (area of impact not described), control cohort selection, or underlying structural/ functional changes as diffusion MRI and functional imaging were not performed (Shao et al., 2018). For a comprehensive review of sex differences in mTBI, refer to Solomito et al. (2018).

#### 1.4.3 Methodological considerations

A variety of methodological considerations are important to address current knowledge gaps. First, multimodal MRI analysis is key for elucidating the mechanisms behind ocular motor dysfunction following mTBI by comparing structure (white matter; dMRI) to function (gray matter; fMRI) (Hasan et al., 2018). Multimodal imaging renders a more thorough understanding to mTBI pathophysiology, such as cerebral blood flow explanations (e.g., arterial spin‐labelling) for abnormal BOLD signals (fMRI), or FA/MD explanations (DTI) for disrupted functional connectivity (fMRI). Second, in dMRI methodology, there is little consensus as to which diffusion measures are most sensitive to mTBI‐related injury (Asken et al., 2018). There are inconsistent results between FA, MD, AD, RD, across subjects, and time points which hinders specificity to mTBI (Tayebi et al., 2021). Standardization of sequences and analysis workflows for diffusion imaging, fMRI, and eye tracking would facilitate smoother data sharing and encourage more groups to perform meta‐analyses in this area. dMRI measurements are also dependent on accurate tractography (correct calculation of fiber direction per voxel) and region of interest (ROI) placement, particularly when probing the subtle nature of eye movement dysfunction. Additionally, dMRI is highly prone to both rigid body motion and cardiac‐induced brain pulsatility, which can easily corrupt entire diffusion data sets. These factors directly influence the FA and MD measurements. Anatomically constrained tractography and the use of probabilistic (as opposed to deterministic) tractography is a method of improving this issue (Smith et al., 2012), along with fixel‐based analysis which is more fiber‐specific than voxel‐based methods (e.g., FA which is a voxel‐averaged measure and prone to poor interpretability) (Raffelt et al., 2017). Subtle white matter tract abnormalities may also be missed by averaging values across an entire tract. Improvements in diffusion analysis should focus on fixel‐based methods and more accurate dMRI measures (e.g., derived from diffusion kurtosis imaging (Jensen et al., 2005)) to highlight precisely where the damage resides. Third, task‐based fMRI studies comparing eye tracking metrics recorded outside the scanner versus inside the scanner have major implications. Understanding the test–retest relationship is integral to the validity of inside‐scanner versus outside‐scanner data (i.e., whether a patient's prescan eye tracking assessment can substitute for an in‐scan protocol). Although, there is likely no replacement for time‐synchronized eye tracking data to real‐time BOLD activation. If a participant follows the same movements on a screen inside the scanner (displaying a time‐synchronized eye tracking protocol without an eye tracking apparatus) while supervised via video feed to ensure compliance, this would spare fMRI institutions from investing in costly and specialized MRI‐compatible eye tracking equipment, facilitating more research in this area. Fourth, exploring the relationship behind reflexive (basic saccade measures such as velocities) versus more complex saccade tasks (e.g., memory‐guided saccades) will clarify whether eye movement dysfunction is due to structural abnormalities to eye movement pathways, higher cortical control (e.g., cognition, executive function, attention), or a combination of these. Standardization of eye tracking protocols with transparent methods of analysis (including an assessment of test–retest reliability, as mentioned previously) will tackle the current issues of heterogeneous data. Fifth, tighter demographic control of time postinjury, mechanism of mTBI, sex, age, and ethnicity would clarify results, which are mentioned in the Limitations section.

#### 1.4.4 Mechanism of injury

The majority of participants evaluated in these studies were sport‐related mTBI, followed by mixed‐cause civilian mTBIs. Notably, there are no studies evaluating MRI and eye tracking measures following mTBIs induced by blast exposure in military populations. These injuries are common in active service personnel and an objective tool to identify injured soldiers in the field would be of great use to the military (Finkel et al., 2017; Leo & McCrea, 2016) as reviewed in Phipps et al. (2020). It is largely unknown as to how the general mechanism of injury (blast‐related, sport‐related, or civilian mTBI, such as road traffic accidents) affects ocular motor impairment, but current knowledge of blast‐related pathophysiology may reveal clues. One such example is from pressure waves in blast‐induced mTBI, which damages the inner ear and peripheral vestibular sensory organs (semicircular canals and otolith organs) (Kerr, 1980). These pressure waves will also damage brain parenchyma from the transmission of kinetic injury, generating diffuse injury in both gray and white matter which leads to a cascade of detrimental neuro‐inflammatory effects (Ng & Lee, 2019). Therefore, classification by physical mechanism such as direct impact loading and/or indirect, inertial, noncontact loading may further assist in exploring this relationship (Gennarelli, 1985). However, these are not mutually exclusive so a combination of detailed history and neuroimaging is required for the most accurate characterization of injury. Therefore, understanding whether relationships between eye tracking and MRI are different depending on mechanism of injury is critical. Post et al. (2019) made the first attempt to describe this relationship from a biomechanical perspective, but their study only accounted for the first phase of mTBI pathophysiology (mechanical injury), not the secondary neurometabolic cascade, nor damage to gray matter structures (only DTI was included). Future studies including the use of accelerometers worn during the time of injury (e.g., contact player cohorts with helmet or mouthguard accelerometers) would allow for more accurate characterization of these forces and subsequent ocular motor dysfunction.

Overall, portable and inexpensive eye tracking technology with automated analysis as a medical diagnostic device appears plausible if this approach can be validated with further research. Current lines of focus should comprise of universal agreement on the most sensitive eye tracking paradigms followed by correlation with multimodal advanced MRI. This work should focus on the predictive value of symptom (or “biomarker”) recovery which would benefit from following patients throughout their rehabilitation in mTBI clinics. Comparing group outcomes in different types of rehabilitation methods (including no rehabilitation) would lead to more objective evidence in this area which is currently lacking. These fields are rapidly progressing and this is likely on the horizon.

### 1.5 Limitations

Across the studies correlating eye movement abnormalities to neuroimaging findings, cohorts are largely heterogeneous in both demographics and time since injury. Common limitations comprise cohort size, participant selection (mTBI severity/repetitive impacts), longitudinal design, issues with translatability, and differences in analysis.

Larger cohorts allow for subgroup analysis of symptom scores, degree of eye tracking dysfunction, and neuroimaging abnormalities. This may prevent milder injuries (which may present a different pattern of injury) from biasing the results of more severe ones. In addition, few studies report the incidence of multiple impacts, or multiple previous mTBIs in their patients which may further increase recovery time (Baker et al., 2015; Prins et al., 2013; Romeu‐Mejia et al., 2019). Longitudinal cohort studies, particularly ones with baseline assessments preinjury, will further increase the sensitivity of modeling the physiological healing response and subsequent clinical translatability. The main barriers preventing the translation of advanced neuroimaging to clinical practice is time (for the patient and staff) and expense. These studies require immense resources for recruitment, day‐to‐day logistics, data storage considerations, and analysis. Due to the inherent complexity of these methods, expertise is required in analyzing these images to draw meaningful conclusions. Examining fMRI results via regions of interest or voxel‐wise methods assumes this heterogeneous spectrum of injury results in a homogenous pattern of injury which is why this method rarely survives group‐wise analysis in small experimental cohorts (Mayer et al., 2015).

Eye tracking requires specialist training and technology, limiting immediate translation from bench to bedside. In the studies above, eye tracking analysis methods in the diffusion studies were often scarcely reported with in‐house, purpose‐built software generated for the study and not reported in detail. Eye tracking protocols were heterogeneous with little similarity in experimental paradigms. Wider collaboration between these methods will allow for more neuroimaging groups to adopt these measures. Additionally, none of these studies have evaluated the discriminative capacities of eye tracking measures between injured/noninjured groups. This is likely due to issues with sample size, but would add both diagnostic and prognostic value to these techniques.

## 2 CONCLUSION

mTBI forms a spectrum of injury from subtle, often transient symptomology, to prolonged symptoms persisting for months or years. The temporal associations with neuroimaging findings cannot be ignored: the acute (48 h) and subacute (2 weeks) compared to the 1‐month and long term (6 months) findings differ substantially. Clinical recovery is not necessarily associated with radiological evidence of recovery with fMRI and dMRI signals suggesting impairment beyond this period. Clinical evaluation through eye tracking analysis enriches data, correlating structure to function. While our understanding of the relationship between MRI abnormalities and eye movement dysfunction remain in early stages, recent efforts integrating attention and higher cortical functions such as reading, smooth pursuit, or switching between prosaccades to antisaccades, prove more sensitive. Smooth pursuits may show increased variability and are also vulnerable to disruption with higher cognitive loads which correlates to differences in the right inferior frontal gyrus and basal ganglia (Astafiev et al., 2015). Their recovery is not clear with a minority showing gaze error variability years postinjury. Vergence‐based tasks have shown differences in activation in the medial occipital lobe, medial temporal lobe, lingual/parahippocampal region, left middle frontal gyrus, and DLPFC (Rockswold et al., 2019), in addition to decreased activations of specific brain stem motor areas (ocular motor nuclei and superior colliculi) (Tyler et al., 2015).

In diffusion MRI studies, the ACR (Maruta et al., 2010), UF (Maruta et al., 2010; Taghdiri et al., 2018), cingulum (Post et al., 2019; Taghdiri et al., 2018), genu of CC (Manning et al., 2020; Maruta et al., 2010; Ting et al., 2016), and cerebellar peduncle (Maruta et al., 2010; Post et al., 2019) have been shown to be disrupted. Multimodal imaging, albeit limited in the literature, is required to elucidate the relationship between these gray matter and white matter regions to understand between‐network dysfunction. Additionally, neurocognitive measures, patient history, and biomechanical data are key in further correlating the varied clinical presentations, endemic to mTBI, to imaging data. Differential recovery following mTBI (e.g., symptom recovery prior to visuomotor and neuronal recovery) exemplifies the need for accurate biomarkers of injury which will, in turn, lead to more accurate diagnosis, prognostication, rehabilitation, and safe return to sport or work.

## CONFLICT OF INTEREST

The authors declare that there is no conflict of interest.

### PEER REVIEW

The peer review history for this article is available at https://publons.com/publon/10.1002/brb3.2714.

## Data Availability

Original MRI and eye tracking data is not available on request due to ethical and legal obligations to research participants. The MRI diffusion analysis pipeline used to construct Figures 5, 6, 7, 8, 9, 10, 11, 12 is available upon request from the authors.
